# Groundbreaking Technologies and the Biocontrol of Fungal Vascular Plant Pathogens

**DOI:** 10.3390/jof11010077

**Published:** 2025-01-18

**Authors:** Carmen Gómez-Lama Cabanás, Jesús Mercado-Blanco

**Affiliations:** 1 Department of Crop Protection, Instituto de Agricultura Sostenible, Consejo Superior de Investigaciones Científicas (CSIC), Campus Alameda del Obispo, Avd. Menéndez Pidal s/n, 14004 Córdoba, Spain; 2 Department of Soil and Plant Microbiology, Estación Experimental del Zaidín, CSIC, Profesor Albareda 1, 18008 Granada, Spain; jesus.mercado@eez.csic.es

**Keywords:** artificial intelligence (AI), biological control, functional peptides, *Fusarium oxysporum*, genome editing, integrated disease management, microbiome engineering, nanotechnology, RNA interference, *Verticillium dahliae*

## Abstract

This review delves into innovative technologies to improve the control of vascular fungal plant pathogens. It also briefly summarizes traditional biocontrol approaches to manage them, addressing their limitations and emphasizing the need to develop more sustainable and precise solutions. Powerful tools such as next-generation sequencing, meta-omics, and microbiome engineering allow for the targeted manipulation of microbial communities to enhance pathogen suppression. Microbiome-based approaches include the design of synthetic microbial consortia and the transplant of entire or customized soil/plant microbiomes, potentially offering more resilient and adaptable biocontrol strategies. Nanotechnology has also advanced significantly, providing methods for the targeted delivery of biological control agents (BCAs) or compounds derived from them through different nanoparticles (NPs), including bacteriogenic, mycogenic, phytogenic, phycogenic, and debris-derived ones acting as carriers. The use of biodegradable polymeric and non-polymeric eco-friendly NPs, which enable the controlled release of antifungal agents while minimizing environmental impact, is also explored. Furthermore, artificial intelligence and machine learning can revolutionize crop protection through early disease detection, the prediction of disease outbreaks, and precision in BCA treatments. Other technologies such as genome editing, RNA interference (RNAi), and functional peptides can enhance BCA efficacy against pathogenic fungi. Altogether, these technologies provide a comprehensive framework for sustainable and precise management of fungal vascular diseases, redefining pathogen biocontrol in modern agriculture.

## 1. Introduction

The effective control of plant diseases represents a significant challenge to global agriculture of the 21st century, exacerbated by the burgeoning world population and the continuous demand of safe food. Moreover, production loses caused by diseases should be minimized under sustainability criteria. With the world population projected to reach 9.7 billion in 2050 [1], food demand will reciprocally increase, requiring more efficient schemes of agricultural production. However, traditional and emergent plant diseases caused by fungi, bacteria, viruses, parasitic plants, and nematodes undermine agricultural efforts worldwide. These diseases not only cause substantial yield losses, which are estimated to range from 20% to 40% annually [2], but also compromise the quality and safety of agricultural products [3]. Data from the Food and Agriculture Organization (FAO) indicate that the economic impact of plant diseases and pests on annual global agricultural production exceeds USD 220 billion [4].

Plant disease management has mostly relied on the use of chemical-based pesticides and fungicides. Although chemicals have proven effective in mitigating disease outbreaks, their abuse and inappropriate utilization have entailed undesirable consequences such as increased environmental pollution (soil, water, flora), the development of pathogen resistance to pesticides, an important loss of biodiversity, and negative effects on human and other animals’ (e.g., bees and other beneficial arthropods) health [5,6]. Nearly 2 million tons of pesticides are used globally to confront plant diseases and pests to maximize agricultural production [7]. In this regard, the European Commission launched the “Farm to Fork” strategy, which in line with the European Green Deal aims to promote healthy and sustainable food systems and restore nature. This plan establishes specific objectives, such as reducing the use of pesticides (by 50%), fertilizers (by 20%), and antimicrobials in livestock and aquaculture (by 50%). In addition, it aims to reconvert into organic production a quarter of the land currently devoted to agriculture. Crucial additional goals are the protection of pollinators and conservation of biodiversity.

The fulfilment of the previous objectives and prerequisites must be ascertained through innovative and sustainable (green) technologies that integrate, among other approaches, plant and microbial resources. This has led to a paradigm shift in current agriculture trends, more focused on the search for environmentally friendly, integrated disease management strategies in which biocontrol plays an important role in order to achieve a reasonable reduction in the use of chemicals. Additionally, the intensification of agronomic practices, fuelled by factors such as population growth, changing dietary habits, and economic development has led to the proliferation of monoculture systems and the expansion of agricultural land into previously untouched areas.

Alterations (e.g., increasing temperatures, modifications of rainfall patterns, extreme weather events, etc.) induced by a climate change scenario can promote suitable conditions for the emergence and spread of new plant pathogens [8,9]. Furthermore, human activities, including urbanization, deforestation, and the globalization (goods and people) process, contribute to pathogens’ dispersal and disturbance of natural ecosystems, making it easier the establishment of novel disease complexes [10,11]. Global trade, above all, allows invasive/exotic species to overstep their natural distribution ranges, further hampering the implementation of effective disease management efforts. It should be emphasized that human-mediated pathogen transport occurs unpredictably and at a large scale, unlike natural transport that follows a regular and expected colonization pattern (based on the dispersive capacity of the organism) [11,12].

In this scenario, unravelling the complexity of plant–microbiome interplay, which includes plant–pathogen, plant–beneficial microorganism, and microbe–microbe interactions, poses a great challenge. Plant pathosystems are dynamic and multifaceted, influenced by factors such as host genetics, environmental conditions, human interventions, and the microbial diversity present in any given agroecosystem. It is then when the concept of plant holobiont (i.e., a meta-organism composed of the macroscopic host and the microbial communities living on/in it and who have co-evolved to shape a delicate ecological entity; [13,14,15]) emerges and acquires utmost relevance. These plant-associated microrganisms are of vital importance for the health and well-being of the host, as they contribute decisively to its growth, development, and ability to confront a range of environmental and biotic stresses, including those caused by the attack of pathogens [15]. It should be emphasized that climate change and anthropogenic actions may alter the distribution and abundance of pathogens and their vectors, as well as that of specific microorganisms within the plant microbiome, affecting disease epidemiology and host susceptibility [16]. Moreover, the widespread use of pesticides and fungicides not only may promote resistance-breaking events in many pathogens, but also can negatively impact the structure and functioning of the microbial communities, posing a threat to global food security and underscoring the urgent need for novel disease control strategies [17,18].

**Figure 1:**
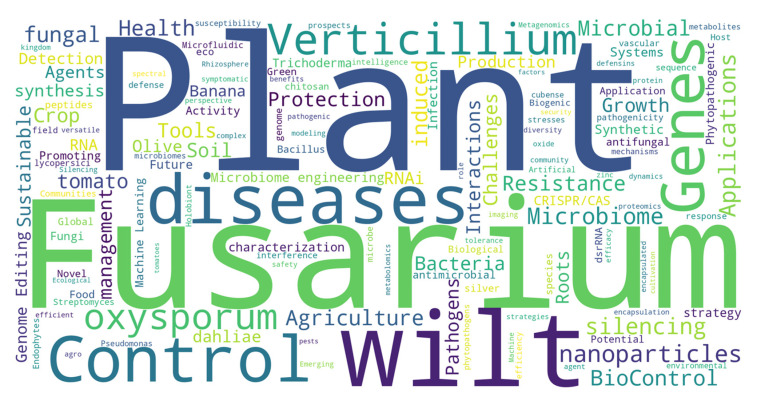
Word cloud showing the most relevant terms extracted from the titles of the articles consulted to produce this review. The frequency each term appears in the titles is visually emphasized in the cloud by their size. The figure was generated using the free online ChatGPT (https://chatgpt.com/, accessed on 12 December 2024).

Agriculture greening and Agriculture 4.0 [19], along with the agrotechnological advances in which this conceptual framework relies, have the potential to accelerate the achievement of some Sustainable Development Goals and the European Commission Green Deal. This article aims to overview innovative approaches on biocontrol in order to optimize integrated disease management strategies, increase precision and effectiveness of biocontrol methods, enhance crop productivity, and decrease the use of chemical fungicides. We will review how current groundbreaking technologies can revolutionize biocontrol strategies to manage diseases, focusing on fungal vascular pathogens (Figure 1). Approaches based on “-omics”, plant microbiome research, nanobiotechnology, artificial intelligence (AI), and other technologies such as genome editing (GE), interference RNA interference (RNAi) and the use of functional peptides will be highlighted. First, we will briefly summarize the relevance of vascular fungal diseases and traditional (bio)control approaches to manage them.

## 2. Relevance of Vascular Fungal Pathogens and the Challenge of Their Control

**Figure 2:**
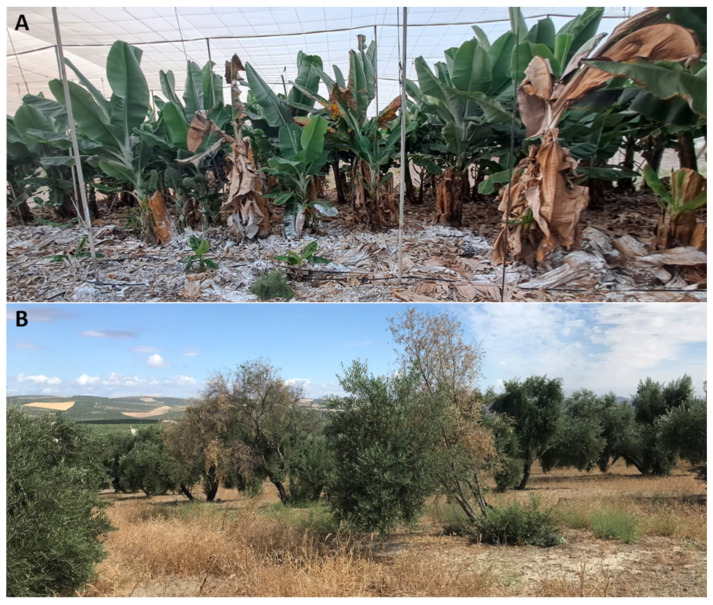
Two examples of fungal vascular diseases affecting highly relevant crops. (**A**) Banana orchard in Tenerife island affected by Fusarium wilt (*Fusarium oxysporum* f. sp. *cubense*) (photo credit Javier López Cepero); (**B**) Olive trees in Southern Spain showing Verticillium wilt (*Verticillium dahliae*) symptoms (photo credit Jesús Mercado-Blanco).

Vascular fungal pathogens are among the most destructive plant pathogens due to their ability to colonize the host vascular system and spread systemically. On average, crop losses due to vascular diseases can range between 10% and 50% of the total production of the affected crop. For example, 10–80% losses have been reported in tomatoes affected by Fusarium wilt, depending on the *Fusarium* strain and climatic conditions [20]. Important production losses caused by banana wilt diseases are also reported globally over time. The diseases are hard to eradicate once established, causing global area loss of up to 1.7 million hectares what represents 17.7% of current land devoted to banana production [21]. Since part of the life cycle of these pathogens (i.e., the parasitic phase) occurred inside the host plant, their effective management is a complicated task. For instance, accessibility and efficacy of many conventional antifungal treatments acting by direct contact with their targets are hindered due to the inner localization of the pathogen. Additionally, some of them are released directly by vectors (e.g., *Ophiostoma ulmi* and *Ophistoma novo-ulmi* transmitted by *Scolytus* spp.) into the plant xylem [22,23]. Additional reasons making it difficult the control of these fungi are: (i) systemic host colonization; (ii) production of resistance structures (e.g., chlamydospores, microsclerotia) that persist in the soil for long periods even in the absence of hosts; (iii) high genetic variability and ability to overcome host resistance mechanisms; and (iv) broad host range, which further complicate management strategies as crop rotation [24]. This group of pathogens includes genera like *Fusarium*, *Verticillium*, and *Ceratocystis*, which cause significant diseases such as Fusarium and Verticillium wilts (Figure 2) or Dutch elm disease. Once inside the vascular system, fungal toxins and biomass (spores, mycelium) together with tyloses, gums and gels produced by the host (i.e., plant defence responses) block the xylem vessesls and disrupt water flow, leading to symptoms such as wilting, yellowing of leaves, stunted growth and eventually plant death [24,25,26,27,28,29,30].

Effective control of these fungi requires the implementation of integrated pest management (IPM) strategies (see, for instance [31,32] which include the use of resistant crop varieties (the most effective method), biological control agents (BCAs) such as beneficial fungi and bacteria with capacity to outcompete or antagonize the pathogens, and cultural practices like improving soil health and drainage to reduce fungal colonization. Advances in molecular biology are also very helpful and relevant, as for example the identification of key virulence genes in vascular fungi that could serve as targets for genetic resistance or for the development of biopesticides [33,34,35]. Despite these efforts, the effective and sustainable management of vascular fungi remains as a major challenge, underscoring the need for innovative and more holistic approaches to protect agricultural systems from the threat posed by these devastating pathogens.

### Traditional Biocontrol of Vascular Fungal Pathogens: A Brief Overview

Biological control of vascular fungal diseases, such as Fusarium and Verticillium wilts or cacao wilt caused by *Ceratocystis fimbriata*, has traditionally involved various approaches, primarily utilizing antagonistic microorganisms and/or their metabolites. One of the most successful and widely studied BCAs are *Trichoderma* spp., particularly *T. harzianum* and *T. atroviride*, which have demonstrated to be effective by producing hydrolytic enzymes (chitinases, glucanases) that degrade the fungal cell walls, and also through direct competition for space and nutrients in the rhizosphere [36]. Additionally, *Trichoderma* is able to induce systemic resistance in plants, which enhances the plant’s innate immune responses to vascular fungal pathogens [37].

Other relevant biocontrol microorganisms are plant growth-promoting rhizobacteria (PGPR) such as *Pseudomonas* spp., *Bacillus* spp. or *Paenibacillus* spp. [38,39,40,41,42,43,44,45]. These bacteria may suppress vascular pathogens through multiple mechanisms like production of antibiotics, siderophores that deprive pathogens of iron, and volatile organic compounds able to inhibit fungal growth [46,47,48]. They are also capable to trigger Induced Systemic Resistance (ISR) and/or Systemic Acquired Resistance (SAR) mechanisms, as well as induce a physiological state of the plant host called “priming”, reducing the severity of infections [49,50]. Similarly, *Streptomyces* spp., have been successfully used to control Verticillium and Fusarium wilts by producing antifungal compounds and cell wall-degrading enzymes, or through hyperparasitism on pathogenic fungi, plant growth promotion and ISR [28,51,52,53].

Treatment based on non-pathogenic strains of *Fusarium oxysporum* isolated from soil and/or stems of healthy plants has also been explored as a biocontrol strategy. Indeed, these non-pathogenic strains colonize plant roots and compete with pathogenic strains for infection sites and resources, effectively reducing the incidence of Fusarium wilt in crops like tomatoes and bananas [54,55,56].

Mycoparasitic fungi such as *Clonostachys rosea* (sym. *Gliocladium roseum*) have been applied to control *Verticillium dahliae* by parasitizing its mycelium and microsclerotia, the latter one being the key survival structures of the pathogen [57]. Rizk et al. [58] also reported biocontrol of *F. oxysporum* in mint by *Gliocladium virens*. The role of Arbuscular Mycorrhizal Fungi (AMF) in biocontrol of Verticillium and Fusarium wilts has also been recently highlighted. Certainly, *Rhizophagus irregularis* and *Funneliformis mosseae* are the most widely used AMF to control these vascular diseases [59]. Likewise, *Glomus intraradices* as well as others *Glomus* spp. have been amply described to increase resistance to soil-borne, vascular pathogens as well as to promote nutrient uptake and modulate the plant immune system thereby contributing to enhance plant health [59,60,61].

All these BCAs can be used individually or in combination as microbial consortia to provide a more comprehensive and resilient strategy against vascular fungal diseases. Different studies have reported that higher disease suppression is achieved when a consortium is used compared with the effect obtained with a single strain. Further, the integration of multiple BCAs that function through different mode of actions offers a sustainable alternative to chemical fungicides, especially in organic farming systems [62,63].

## 3. Leveraging Meta-Omics for Biocontrol Optimization

Metabarcoding and metagenomics, together with other “meta-omics” disciplines such as metatranscriptomics, metaproteomics, and metametabolomics, are powerful and multifaceted tools with huge potential in the (bio)control of fungal vascular diseases. Metagenomics has been essential in the accurate and rapid culture-independent detection and identification of vascular pathogens [64]. Metagenomics also provide a comprehensive view of plant–microbe interactions [65]. Moreover, it allows monitoring soil and plant health, detecting changes in microbial communities that could predispose plants to infections or, on the contrary, to show greater resistance to them. Metagenomics data can also facilitate the development of biological control strategies by identifying antagonistic microorganisms and generating microbial consortia with enhanced suppression ability of plant pathogens [66]. By using these approaches, a more complete picture of the composition and structure of the microbial communities present in the plant vascular system is obtained, thereby enabling the identification of indigenous beneficial microorganisms that could act as pathogen antagonists [44]. Finally, these potent tools are valuable for environmental surveillance and crop monitoring, helping to predict and prevent outbreaks of fungal vascular diseases through the early identification of pathogens or by pinpointing changes in microbial communities under specific situations [67,68].

Metatranscriptomic approaches are useful to study microbial communities and plant gene expression in response to infections. This makes it easier to understand how pathogens cause diseases and how plants and their microbiomes respond to these attacks. Focusing on vascular pathogens, a metatranscriptomic approach unravelled the dynamics of the rhizosphere microbiome in olive plants after *V. dahliae* infection [69]. Results from this study suggested that Verticillium wilt is not only driven by *V. dahliae* but rather by a polymicrobial consortium that also includes natural microorganisms of the olive root endophytome.

Metaproteomics also provides a better understanding of the function, structure, dynamics, and significance of plant-associated microbial communities. It has been very helpful to investigate protein profiles expressed by both microbial communities and plants during their interactions [70]. Metaproteomic analyses can reveal the functional relationships of plant–microbe and microbe–microbe interactions under specific environmental conditions. Basically, the metaproteome offers the opportunity to better understand the microbiota composition and dynamics, as well the metabolism and physiology of the (micro)organisms involved, including the host. This “omic” is also fundamental for deciphering soil fertility, nutrient cycling, and bioremediation. Although metaproteomics applied to plants has progressed in recent years, some technical challenges (e.g., improving the effectiveness and specificity of protein extraction procedures) hampering a broader implementation of this approach still need to be addressed to study, for instance, the root/vascular-associated microbiota [71].

Finally, metabolomics is also a useful tool for understanding plant–microorganism interactions, particularly the identification and analysis of bioactive compounds produced during these complex interactions. Metabolomics offers valuable information on changes in metabolic profiles associated with plant pathogenesis that can affect plants, phytopathogens, and/or beneficial microbes [72,73]. As an example for our topic, Hu et al. [74] demonstrated differential responses in tomato–*V. dahliae* compatible and incompatible interactions through metabolome analyses, unveiling biochemical pathways associated with plant (a)biotic stress that can contribute to improve biocontrol methods.

## 4. Microbiome-Driven Approaches and Biocontrol

The huge advances in “omics” tools in recent years have drastically increased our knowledge on plant microbiomes, enabling the identification of links between them and diseases, as well as to explore new avenues for their control. Current research highlights the close, often symbiotic, relationship existing between microorganisms and plants. This association governs, at least to some extent, plant growth, promotes resilience to different a/biotic stresses, and improves the general plant fitness [75]. As mentioned above, plants and their associated microbiota (a myriad of bacteria, fungi and other eukaryotes, archaea, and viruses) constitute holobionts [14,15,76]. Beneficial interactions are responsible for maintaining the holobiont’s health, while diseases are often correlated with microbial dysbiosis or imbalances in the holobiont assemblage [77]. Microbial diversity was identified as a key factor in disease prevention and can be implemented as a biomarker in plant protection strategies. The objective would be to apply targeted and predictive biocontrol approaches by developing microbiome-based solutions which can be included in breeding programs and combined with other control methods. Understanding the plant microbiome has led to a paradigm shift in our understanding of its important role in health and disease, with substantial consequences for biocontrol strategies [78]. Thus, the manipulation or customization of plant microbiomes for enhancing plant growth and protection against stresses constitutes a research avenue with great potential [79,80].

### Microbiome Engineering

The last few years have witnessed intensified efforts to exploit microbial communities for disease control. This is particularly true for microorganisms inhabiting the rhizosphere [81,82], the phyllosphere [83,84], or the plant interior (i.e., endophytes) [85,86]. Beneficial microbes present in these niches may then confer resistance to diseases through different mechanisms such as competition for space or nutrients, antibiosis, and ISR. At the same time, high-throughput sequencing technologies, along with advances in bioinformatics and increasing data analysis capabilities, have revolutionized plant pathology by allowing researchers to elucidate the genetic basis of interactions between plants and microorganisms (including pathogens) and to discover the molecular bases underlying disease onset and progress and host resistance. However, the amount and the complexity of continuously generated multiomics data pose significant challenges in terms of storage, management, and analysis [87,88,89].

Plant microbiome engineering involves the deliberate manipulation of plant-associated microbial communities to improve growth through nutrient uptake and disease resistance under stress [90]. Understanding this manipulation process will be instrumental for the optimal design of next-generation microbial inoculants aimed at disease suppression and plant growth promotion [91]. Microbiome engineering contemplates both the direct inoculation of exogenous beneficial microorganisms and the re-inoculation of autochthonous beneficial microorganisms enriched ex situ [92]. Two distinct approaches have been developed in plant microbiome engineering. On the one hand, bottom-up approaches involve the isolation of microorganisms whose physiologic features have been well characterized. Co-cultivation trials are carried out to understand and identify pairwise interactions among these strains. Additionally, systems based on cell-to-cell communications are employed to directly control the behaviour of specific populations. On the other hand, top-down methods start with the selection of an effective microbiome (seeding microbiome) containing unculturable microorganisms. This approach involves synthetic ecology, which includes horizontal gene transfer to a variety of hosts in situ and then phenotyping the microbiome. Three methods have been proposed in the top-down strategy: enrichment, artificial selection, and directed evolution [93,94]. Enrichment involves the introduction of the seeding microbiome into several growth–dilution cycles under precisely controlled environmental conditions. It is expected that the microbiome would progressively adjust to the surroundings thereby achieving greater performance. In the artificial selection strategy, a set of low-density ‘newborn’ communities is allowed to mature (i.e., to become ‘adult’ communities) in a predetermined period of time. The next cycle is then initiated by selecting the adult communities with improved functionality to generate a new generation of newborn communities. Finally, directed evolution commences by building a library of stable (i.e., throughout generations) communities displaying a range of functions. The community that shows the greatest function is then exposed to ecological disturbances. Eventually, and to begin a fresh iteration, a new library of generationally stable communities is generated [93].

In addition, two other approaches to engineer the rhizosphere microbiome are emerging: (i) prebiotics made from specific plant root exudates to attract and maintain a beneficial microbiome [95] and (ii) crop breeding for improved beneficial microbiome interactions [96]. For example, a Fusarium-resistant bean cultivar can selectively recruit beneficial *Paenibacillus* bacteria in the rhizosphere, which enhance disease suppression by upregulating genes involved in the production of antimicrobial compounds such as phenazine and colicin V, thereby protecting the plant against *F. oxysporum* f. sp. *phaseoli* [97] (Table 1).

Another promising microbiome-based strategy for controlling plant pathogens is microbiome transplantation [91,158]. To our knowledge, no reports on the implementation of this approach to control vascular fungi are available. So far, studies have just focussed on microbiome differences observed between plant genotypes that are either tolerant or susceptible to these pathogens (e.g., [97,159]). However, the transplant of rhizosphere microbiota from tomato plants resistant to *Ralstonia solanacearum*, a destructive vascular pathogen causing bacterial wilt, was shown to be effective in suppressing disease symptoms in susceptible plants [160]. Additional examples of success using this strategy have also been reported for nonvascular fungal pathogens [161], which allows predicting its implementation to manage vascular diseases caused by fungi.

Our capacity to design formulations based on single microbes, consortia, or synthetic communities (SynCom) and customized microbiomes for biocontrol and biofertilization is being enhanced by potent technologies like the ones summarized in this review. Tools to simulate or predict the performance of modified microbiomes prior to their assessment under field conditions have thus been developed. For example, tiny microscopic containers for organizing many bacterial species in almost any 3D geometry may now be produced thanks to lithographic 3D printing [162,163]. This allows unveiling and comparing their interactions with native strains while examining them at the microscopic level. Engineered microbiome can also benefit from the use of a “tracking root interactions system” (TRIS), a microfluidic device designed to monitor interactions between bacteria and roots [164,165]. More examples of microfluidics chambers designed to study complex interactions between bacteria and plants, such as “RMI-chip”, “Plant on a chip”, “RootChip”, “RootArray”, “TipChip”, “PlantChip” ([166] and references therein), and static droplet array [167,168], have been reported. These microfluidic platforms show great potential for advancing targeted control methods against vascular fungal pathogens in plants and would allow for the precise manipulation of infection conditions and pathogen–host interactions [169,170].

Undoubtedly, engineering microbiomes that are more durable and stable over time, resistant to environmental stressors, and capable of increasing agricultural production will be instrumental to improve biocontrol methods in the future [171].

## 5. Nanotechnology and Biocontrol of Plant Fungal Vascular Diseases

Combining biotechnology and nanotechnology to improve the effectiveness and sustainability of plant disease management may lead to advances at an unprecedented scale. Indeed, the combination and synergies of both technologies (i.e., nanobiotechnology) has shaken up plant disease management strategies. Nanotechnology involves the study, design, production, and usage of molecules, compounds, structures, devices, and systems at nanoscale (size between 1 and 100 nm) and has interesting applications in agriculture [172]. The integration of nanoparticles (NPs) in biocontrol strategies represents a significant (r)evolution in this area. NPs offer unparalleled promising solutions to address difficulties inherent to the biocontrol of plant vascular diseases. Their unique physicochemical properties enable greater efficiency and penetration capability. For instance, they can be transported directly to plant vascular tissues under schemes of controlled release, reducing toxicity and side effects. Moreover, they show compatibility with other control strategies and adjust to sustainability criteria [173,174,175]. Some of the nanomaterials used in plant diseases control are alginate, chitosan, carbon, copper (Cu), gold (Au), magnesium (Mg), silica (Si), silver (Ag), titanium (Ti), and zinc (Zn) [176]. In this review, we will focus specifically on antifungal NPs compatible with biological control, especially those synthesized through environmentally friendly or ’green’ methods. We will also review certain natural and biodegradables nanomaterials, some of them with inherent antifungal properties that also serve as carriers of BCAs, compounds derived from them, or other natural substances able to combat fungal vascular pathogens (Table 1).

### 5.1. Nanoparticles Biosynthesis and Potential Use in Biocontrol

The synthesis of NPs by biological methods opens new avenues for the production of these materials using natural reducing and stabilizing agents [177]. Nature provides eco-friendly precursors or biofactories for the synthesis of metallic NPs and metallic oxide NPs [178,179,180]. Among them, bacteria [181], fungi [182], yeasts [183], viruses [184], algae [146], plant extracts [185], and waste materials [186] have been described in the literature. It is an economical and sustainable alternative to chemical and physical procedures with reduced inputs of energy and toxic chemicals. In spite of these advantages, the biological synthesis of NPs frequently requires time-consuming cultivation of microorganisms, among other drawbacks [187]. Nonetheless, the heedful selection of microorganisms, careful control of factors such as pH and temperature, and precise concentration of precursors may allow the scaled production of NPs. In addition, since microbes can be genetically engineered, larger control over the shape and size of NPs, factors that will determine their properties, can be achieved. Ongoing studies have uncovered new capabilities of microorganisms with unprecedented potential to produce NPs. Yet, the exact underlying mechanisms of biogenic synthesis are not fully understood [187]. We will briefly overview different NPs according to the source from which they originate.

#### 5.1.1. Bacteriogenic Nanoparticles

Bacterial cells are stores of numerous metabolites, being able to reduce metal ions to their corresponding NPs that are subsequently stabilized. The synthesis of NPs by bacteria offers advantages compared to that of other biofactories since they are easy to maintain, produce high yields, and pose low purification costs [188]. The synthesis of metal and metal oxide NPs in bacteria (and in other microorganisms) may take place either intracellularly or extracellularly. Intracellular synthesis involves the absorption of metal ions and the subsequent transformation into elemental forms by enzymatic reduction. On the contrary, in the extracellular synthesis, microorganisms produce and release enzymes and other proteins that reduce metal ions and stabilize the NPs [189]. For example, Gopinath and Velusamy [106] reported the synthesis of AgNPs using the supernatant of *Bacillus* sp. GP-23 cultures. The bioreduced AgNPs showed an inhibitory effect on the hyphal growth of *F. oxysporum*. Interestingly, greenhouse application of AgNPs mediated by *Bacillus amyloliquefaciens* MH046937 showed a considerable biocontrol effect against the wilt and root-rot pathogens *F. oxysporum*, *F. solani*, and *R. solani*. The growth stimulation of *Vicia faba* plants compared with plants treated with a chemical fungicide was observed as well [190].

#### 5.1.2. Mycogenic Nanoparticles

The synthesis of NPs using fungi has also received increasing attention due to advantages such as easy scaling up and downstream processing, economic feasibility, simplicity, increased surface due to the presence of mycelia, and simple maintenance [136,188]. In fact, fungi are considered as the most efficient biotechnological factories [136]. Numerous fungal species are suitable candidates to biosynthesize metal NPs (e.g., AgNPs), both intracellularly and extracellularly, being truly efficient due to their high tolerance to metals. Furthermore, fungi produce many extracellular proteins that aid the reduction of metals to NPs. Among them, different strains of *Fusarium*, *Aspergillus*, *Verticillium*, and *Pencillium* have been extensively explored as prospective sources for the production of NPs [191]. For example, many *Fusarium* species were screened to identify promising candidates, *F. oxysporum* being the species producing the smallest size of AgNPs [192]. Biogenic AgNPs synthetized by the phytopathogenic fungus *Alternaria* sp. showed in vitro antifungal activity against *F. oxysporum*, as well as against *Alternaria* sp. itself [107]. Likewise, AgNPs biosynthetized by *T. asperellum* were found to be highly effective in suppressing the mycelial growth of four soil-borne pathogens (*viz.*, *Rhizoctonia solani*, *F. oxysporum*, *Sclerotinia sclerotiorum*, and *Sclerotium rolfsii*) compared to the effect caused by the systemic fungicide carbendezim. The biosynthetized AgNPs by *Asperigillus nigrun* inhibited the growth of three different pathogenic fungi, including *F. oxysporum, Aspergillus flavus*, and *Penicillium digitatum* [108]. El-Sayed et al. [136] demonstrated the effectiveness of biogenic Co_3_O, CuO, Fe_3_O_4_, NiO, and ZnO NPs synthetized using the endophytic fungus *Aspergillus terreus* against Fusarium wilt in *Phaseolis vulgaris*. These biogenic NPs also enhanced the growth of beans under greenhouse conditions.

#### 5.1.3. Phytogenic Nanoparticles

Several plants have the capability to accumulate metals and transform them into NPs intracellularly [188]. Plant extracts are considered advantageous for NP synthesis due to their availability, renewable nature, simplicity of the process, efficiency, rate and stability of synthesized NPs, and cost effectiveness [193]. Studies in extensively grown crops have shown that biosynthesized NPs of titanium dioxide (TiO_2_) applied on wheat plants have potent fungicidal activity and show effective control of *Bipolaris sorokiniana* and *Puccinia striiformis* f. sp. *tritici* [194,195]. Some NPs can trigger SAR against pathogens [196], as shown by Karmous et al. [197], who reported that Zn and Cu oxide NPs can strengthen soybean resistance against *Fusarium virguliforme*. Currently, however, only a few studies investigating the use of phytogenic NPs to combat vascular fungi are available. For instance, Ashraf et al. [132] demonstrated that Cu oxide NPs (CuO-CFNPs) produced using leaf extracts of *Cassia fistula* exhibited antifungal activity against *F. oxysporum* f. sp. *lycopersici* by boosting growth and defence response in tomatoes. Moreover, some phytogenic NPs have been demonstrated to enhance plant growth and function as nanofertilizers [198,199].

#### 5.1.4. Phycogenic Nanoparticles

Algae can accumulate metals and reduce metal ions. This property makes them an inexhaustible raw source for NP biosynthesis. Furthermore, algae can be easily manipulated, allowing low-temperature synthesis with high energy efficiencies, low toxicity, and little environmental risk. Metallic NPs can be synthesized from algae biomass regardless of whether or not they are alive, which poses additional advantages as biofactories [200]. A number of studies have reported that marine algae extracts contain various phytochemicals with amino, sulphate, carboxyl, and hydroxyl functional groups acting as catalysts of precursor metal salts, which are subsequently reduced to nucleated NPs [201,202]. Iron (Fe), Ag, ZnO, TiO_2_, Au, cadmium (Cd), and palladium (Pd) NPs have been biosynthesized by members of the Chlorophyceae, Phaeophyceae, Rhodophyceae, and Cyanophyceae families. Algae-based NPs have been successfully used in medicine and environmental sciences, which has paved the way for their introduction in agriculture, particularly in crop protection [203]. Silver nanobioparticles synthesized from extracts of the *Padin pavonica* (Phaeophyta) thallus inhibited the growth of two important cotton pathogens (*F. oxysporum* f. sp. *vasinfectum* and *Xanthomonas campestris* pv. malvacearum) [146]. Recently, *Xanthomonas citri* pv. citri, the causal agent of citrus canker disease, has been inhibited in vitro using biologically active AgNPs and CuNPs synthesized from macromolecules extracted from *Oedogonium* sp. [204]. Nevertheless, only a few algae-based NPs have been used to control plant diseases so far [203], and more specifically, vascular pathogens.

#### 5.1.5. Nanoparticles Derived from Waste

Another approach of synthesizing NPs is the extraction of active ingredients from agricultural waste products and their transformation into nanoforms [205]. For instance, T-Thienprasert et al. [206] synthetized ZnO NPs from banana peels using a green chemistry method. They showed these NPs displayed inhibitory effects against *Colletotrichum* sp. strain KUFC 021, a fungal pathogen causing anthracnose in orchids, by reducing pathogen growth and disease symptoms. Likewise, SiO_2_ NPs synthetized from sugarcane bagasse and corn cob in vitro inhibited *F. oxysporum* and *Aspergillus niger*. In addition, these NPs had a favourable effect on the growth and germination of *Eruca sativa*, enhancing plant production [207]. Despite these promising results, examples of NPs derived from waste for controlling vascular fungal pathogens in plants are unknown to us.

### 5.2. Nanoparticles as BCA Protectants and/or Carriers

Recent accomplishments regarding nanomaterial synthesis and characterization techniques have expanded even further the repertoire of nanobiotechnology-based solutions for plant disease management. Researchers are leveraging the power of nanotechnology to discover more sustainable and ecological approaches in crop protection, ultimately contributing to improving agricultural sustainability and global food security [208].

It is not unusual that BCAs show inconsistent performance under field conditions [209]. Interestingly, advances in the encapsulation of microorganisms in NPs have been achieved in response to the growing need for more efficient and consistent BCAs. The development of sustainable, NP-based systems to release BCAs (or their bioactive compounds) may increase their efficiency and mitigate potential negative impacts on the environment. The improved efficacy of BCAs can be achieved by prolonging their shelf life, controlling their release to the target niche, and maintaining their metabolic activity during storage, thus reducing the dosage and number of applications. The controlled and slow release of the active molecules relies on the degradation properties of the nanocarrier (NC) (e.g., polymers), the binding capacity of the ingredients to the material, and the environmental conditions (pH, light, temperature, leaching). Furthermore, encapsulation in NPs can improve the adhesion of BCAs to plant surfaces, facilitating more effective and prolonged colonization of the host. This is crucial to ensure that BCAs remain at the site of action, where they can compete with pathogens for nutrients and space, producing bioactive compounds against them and promoting plant growth.

Some NC only transport active ingredients into the target, while others also function as active compounds synergistically complementing and enhancing the antimicrobial activity of the BCA. This synergistic effect may be particularly useful in cases where pathogenic fungi have developed resistance to conventional treatments. The combination of the direct action of NPs and the control mechanisms deployed by BCAs can overcome the pathogen’s defences, offering a more robust and long-lasting solution. The most suitable NPs used as carriers for the delivery of biopesticides are based on polymers (soft NPs), synthetic silica, titania (TiO_2_), alumina (Al_2_O_3_), Ag, Cu, and natural minerals/clays with nanoscale dimensions (inorganic or solid NPs) [210]. According to Hudson and Margaritis [211], 20 techniques for the elaboration of polymeric nanocapsules have been developed. Among them, ionic gelation (extrusion or cross-linking), spray-drying, and emulsion are most often used to encapsulate beneficial microorganisms [212]. Ionic gelation technology consists of dispersing an aqueous solution of sodium alginate (pectinate derivatives or guar gum) containing the desired BCA on a solution containing divalent cations, such as calcium chloride [213]. The spray-drying technique requires dispersing a BCA in a polymer solution that forms an emulsion [214]. Emulsification is a process that involves the dispersion of one liquid (including BCA or their bioactive substances) into another immiscible liquid using emulsifiers to homogenize the mixture [215]. Different NCs, such as nanopolymers, organic and inorganic nanomaterials, and nanoemulsions, can be employed for phytopathological aims. We will now briefly overview some polymeric and non-polymeric nanomaterial commonly used in encapsulation processes.

#### 5.2.1. Encapsulation Based on Nanopolymers

Biodegradable polymeric NCs have attracted significant interest for agricultural research in recent years. These nanomaterials can cross structures such as cell walls and membranes. By passing through plant vessels and transporting active components, which will be of crucial relevance for the pathogens considered in this review, these NCs deliver bioactive products to the target tissue more effectively. Among natural NCs, gelatin, chitosan, plant gums, pectin, starch, and alginate must be mentioned. Chitosan, a polymer derived from chitin, is one of the most widely used in NP formulation for biocontrol due to its antimicrobial properties, biocompatibility, and biodegradability. Chitosan NPs can encapsulate biocontrol microorganisms such as *T. harzianum* or *Pseudomonas fluorescens*, protecting them from adverse environmental factors, increasing their efficacy and releasing them in a prolonged manner in plant roots, which would improve their colonization and ability to fight vascular pathogens such as *F. oxysporum* and *V. dahliae* [216]. For instance, chitosan NPs alone have the potential to suppress the establishment of *F. oxysporum*, and their effect is greater than that of bulk chitosan [109]. Saberi-Riseh and Moradi-Pour [217] encapsulated *Streptomyces fulvissimus* Uts22 using chitosan and gellan gum in order to suppress take-all disease caused by *Gaeumannomyces graminis,* a pathogen capable of invading the vascular system but unable to grow systemically in it. Greenhouse experiments revealed that encapsulation increased the releasing time of the BCA and achieved higher disease suppression. Similarly, *P. fluorescens* cells encapsulated in NPs of chitosan greatly reduced Fusarium wilt in tomato plants [133]. This outcome was attributed to the significant increase in enzymatic activity in the soil due to the controlled release of the BCA. Alginate, derived from algae, is another key polymer in the synthesis of NPs used in biocontrol, with similar behaviour to that of chitosan in terms of biodegradability and biocompatibility. A PGPR consortium (*Pseudomonas* sp. DN 13–01, *Sphingobacterium suaedae* T47, *Bacillus pimilus* X22, and *Bacillus cereus* 263AG5) encapsulated in alginate (extracted from the brown seaweed *Bifurcaria bifurcata*) NPs showed better suppression of Verticillium wilt in tomato than single (PGPR consortium or alginate) treatments. The use of encapsulated PGPRs in this polymer protected the consortium and ensured its gradual release in tomato roots. Moreover, it also improved the plant natural defence response [149].

#### 5.2.2. Encapsulation Based on Non-Polymeric Nanomaterials or Nanoemulsion

BCAs or their bioactive compounds can also be encapsulated in non-polymeric NPs such as liposomes or using nanoemulsions. Liposome NPs consist of vesicles formed by lipid bilayers that can encapsulate both hydrophilic and hydrophobic compounds, making them versatile systems for the release of either BCAs or antifungal metabolites derived from them or from another natural source. In tomato plants, chitinase and laminarinase (from *Trichoderma* sp.) encapsulation in soy lecithin liposomes allowed the control of *F. oxysporum* f. sp*. lycopersici* and significantly promoted plant growth [134]. Atienza et al. [117] synthesized and characterized nanobiofungicides by encapsulating ethanol crude extract from a plant growth-promoting bacterium (*Lysinibacillus fusiformis*) in nanoliposomes. They demonstrated the antifungal activity of the nanobiofungicides against three *formae speciales* (*cubense*, *lycopersici*, and *cucumerinum*) of *F. oxysporum* in in vitro growth inhibition assays. Finally, nanoemulsion has been used for the encapsulation of hydrophobic compounds such as essential oils [218]. Examples such as this one encourage research aimed to develop and use nanoemulsions to combat vascular fungal pathogens, for which no examples are currently available to our knowledge.

## 6. Artificial Intelligence in Biocontrol

Artificial intelligence (AI) is the ability of computer systems to embark on assignments that typically require human cognition, such as learning, natural language processing, pattern recognition, reasoning, visual perception, prediction, and decision-making [219,220]. Basically, AI consists of developing algorithms that allow machines to emulate facets of the human intellect like processing and adapting to information over time. AI covers several sub-fields: machine learning (ML), which identifies data patterns without direct programming [221]; computer vision, which provides machines the ability to ‘understand’ and ‘interpret’ images [222]; and natural language processing, which allows them to comprehend and generate human language [223].

The integration of AI and ML has revolutionized and transformed research fields such as precision agriculture, offering a promising avenue for increasing yields while minimizing negative impacts for the environment. AI also contributes to managing phytopathological challenges through early and rapid disease detection, improved disease diagnostics, and better monitoring and prediction systems [224]. On the one hand, AI-powered platforms continually learn from new data inputs, refining their predictive accuracy and adaptive capabilities over time. On the other hand, ML algorithms can identify complex relationships and patterns within these datasets, providing valuable insights into disease dynamics and contributing to more science-based management practices. AI-driven systems can process and integrate large amounts of data from various sources, ranging from environmental parameters provided by weather stations, agricultural databases, remotely sent images, plant characteristics, and pathogen genetic information. By compiling and computing these data, these systems are capable of establishing disease patterns and forecast outbreaks with high accuracy. This allows farmers to make timely, fast and precise interventions in their crops, optimizing resources (e.g., the use and input of BCAs, fertilizers and water), minimizing pathogen spread, improving plant health, and significantly reducing losses and impact on crop production [224]. The incorporation of AI into the agricultural sector promises to transform disease control, boosting efficiency and adaptability to face present and future environmental and socioeconomic scenarios.

### 6.1. Potential of AI Tools in Biological Control of Vascular Phytopathogenic Fungi

The incorporation of AI in the biological control of plant diseases may represent a revolutionary approach in phytopathology, sustainable agriculture, and food safety (Table 1). AI can assist in the identification and selection of BCAs, as well as in optimizing their usage depending on environmental conditions and other factors that usually affect their consistency and efficacy. Furthermore, AI algorithms would be fundamental in predicting the long-term impact of BCAs, ensuring their effectiveness and safety under diverse ecological contexts. The integration of genomic analysis and bioinformatics with AI can also speed up the comprehension of pathogen resistance mechanisms to facilitate more robust BCA selection. Finally, by simulating plant holobiont–pathogen–BCA interactions, AI would assist researchers in modelling complex dynamics to further improve sustainable disease control strategies. Taken together, these advancements stand out as part of AI’s transformative potential to revolutionize biological control practices, guaranteeing both agricultural productivity and ecological balance within a complex context of growing food demand and climate change challenges [225,226].

### 6.2. Early Detection, Accurate Diagnosis, Risk Prediction, and Infection Modelling

AI, through technologies like computer vision and ML, can significantly improve the early detection of vascular fungal infections. For instance, by using drone imagery or ground-based sensors, AI algorithms can analyse visual patterns in crops, identifying signs of infection at early stages. This may help farmers, among other management actions, to deploy biological control measures more efficiently (e.g., application at the most appropriate time during the infection process) and potentially reducing the spread of disease before critical levels. This could be of relevance for the control of vascular fungi since visible symptoms caused by them (e.g., wilting) already indicate the presence of the pathogen in the xylem vessels, when the disease is at a very advanced stage. Thus, the early detection of Verticillium wilt in potatoes in the absence of visible symptomology using near-infrared spectroscopy and ML modelling [150] highlights one of these innovative applications. Similarly, Selvaraj et al. [118] successfully developed an AI-based detection system for banana diseases (including Fusarium wilt in banana) and pests by using a deep convolutional neural network. Advances like this and others [119,120,123,135,137,138,141,151,152,156,157] demonstrate how these approaches can significantly support farmers in making well-informed management decisions, including the use of biocontrol tools (Table 1).

Likewise, AI-based methodologies can process large datasets related to environmental factors (temperature, humidity, soil conditions, etc.) to predict, for instance, the likelihood of phytopathogenic fungal outbreaks. Predictive models based on ML or neural networks can correlate these variables with fungal growth patterns and disease outbreaks, allowing farmers to apply BCAs at the most favourable times, improving efficacy. In this regard, Blekos et al. [153] developed an intelligent olive orchard monitoring system using multispectral images and computer vision combined with ML techniques. This system helped to predict the spread of Verticillium wilt and provided a decision support system for farmers/agronomists. López-Escudero et al. [154] later proposed a set of “Fuzzy Logic” models [227] as an expert technique to generate a decision support system for Verticillium wilt in olive that was compared with ML models. Similarly, ML models allowed to predict the severity caused by *F. oxysporum* f. sp. *ciceris* in chickpea with reasonable accuracy [121]. Combining these approaches with model combination techniques (integration of multiple predictive models such as Constrained Least Squares or Complete Subset Regression) further enhanced the precision of the prediction.

### 6.3. AI-Assisted Identification, Selection and Optimization of BCA

AI could also assist in optimizing the identification and selection of BCAs against vascular pathogens. However, to our knowledge, no report dealing with the identification and selection of beneficial microorganisms to specifically combat plant vascular fungi using (or assisted by) AI has been published yet. In contrast, examples of the use of AI tools to search for BCAs against other plant pathogens (and pests) are available. Thus, Sadeghi et al. [228] reported the use of ML for the screening of potential probiotic lactic acid bacteria with antimicrobial properties. Also, the semi-automatic identification of phytoseiid mites acting as BCAs against pests such as *Neoseiulus barkeri* Hughes has been possible through an ML approach, namely ‘eXtreme Gradient Boosting’ [229]. Examples like these ones allow us to envisage great potential for AI to identify and characterize the most appropriate antagonists for any given pathosystem. By analysing large datasets from previous research on plant–pathogen–biocontrol interactions, AI can identify the most effective BCAs for a given pathogen and specific environmental conditions, as well as to predict their potential impacts (see Section 6.4). For instance, AI could predict the most appropriate strains of *Trichoderma, Bacillus, Paenibacillus, Pseudomonas*, etc., or combinations (i.e., consortia or SynComs; see Section Microbiome Engineering) of them, to effectively control *F. oxysporum* or *V. dahliae* under specific pedological, climatic and/or agronomic conditions, improving and/or supporting recommendations obtained from empirical data (i.e., [41,42,63,75]).

Once the appropriate BCAs are selected, AI can assist in optimizing their application. ML algorithms can be tailored to calculate the ideal dosage, timing, and distribution of BCAs to maximize their efficacy. By simulating various environmental conditions and reviewing historical data, AI can recommend specific actions, thereby reducing costs and improving biological control performance, ensuring the best usage of these agents. The feasibility of statistical vs. AI approaches, such as ‘Response Surface Methodology’ and ‘Artificial Neural Networks’, to optimize the composition of the culturing medium for high anti-*F. oxysporum* activity of the BCA *Streptomyces* sp. TN71 has been compared. Thus, an increase of almost 60% was obtained using the AI-predicted, optimized medium compared to the original one [110]. Recent advancements also incorporate hyperspectral imaging and infrared thermography combined with ML to optimize the monitoring and real-time inoculation of BCAs. For instance, two strains of *T. harzianum* have been studied for their efficacy against Fusarium wilt in baby lettuce using these technologies [147]. Hyperspectral imaging captures plant physiological changes and spectral signatures, while infrared thermography measures temperature variations indicative of stress or infection. When analysed with ML algorithms, these techniques allow precise monitoring of BCA performance and the environmental conditions that may influence their effectiveness. This data-driven approach enables timely adjustments to optimize BCA application strategies. Interestingly enough, AI approaches can also be used to optimize the biosynthesis of NPs (see Section 5.1) by microorganisms displaying antagonistic effects against plant pathogens [230]. These authors reported an eco-friendly and optimized approach for the biosynthesis of chitosan NPs using *Streptomyces microflavus* NEAE-83 [231] by using an Artificial Neural Network (ANN), a teaching tool applied in ML [232]. Computational methods, including ML and AI, not only can enhance the screening and identification of BCAs, but also improve the design of consortia, SynComs or customized microbiomes making them more stable and effective [233,234]. These technologies can be of great help in, for instance, tailoring SynComs displaying traits for robust colonization, prevalence throughout plant development, and specific beneficial functions for plants [233].

### 6.4. Predicting the Impacts of BCAs

Even though BCAs are more environmentally friendly than synthetic pesticides, they may have undesirable effects. Inoculation with BCAs can have impacts (both direct and indirect) on non-target species, and predicting these effects (especially indirect impacts) remains a core challenge in biocontrol risk assessment. The effect of the introduction of BCAs effective against fungal vascular pathogens on the structure, composition, and co-occurrence networks of plant root-associated microbial communities has been studied through Next Generation Sequence data analysis [235,236,237]. Gómez-Lama Cabanás et al. [236] demonstrated that the well-known BCA *Pseudomonas simiae* PICF7 [45] did not significantly alter either the composition or the structure of the banana root microbiota. However, PICF7 triggered significant changes in the interactions of the microbial community present in this organ. In agreement with these results, Cardoni et al. [237] observed that inoculations with either PICF7 or *Paenibacillus polymyxa* PIC73, another effective BCA against Verticillium wilt in olive [42], caused significant changes neither in the structure nor in the taxonomic composition of the resident olive (cv. Picual) root microbiota. Once again, however, alterations in the topology of the co-occurrence networks were reported. In this regard, AI can go a step further by helping to predict not only the effects (positive, negative, or null) of a single BCA but also those caused by the treatment with microbial consortia or SynComs and, more importantly, to predict the effects that transplants of complete microbiomes (see Section Microbiome Engineering) will have on a target plant holobiont and the surrounding environment. So far, to our knowledge, studies on AI-based predictions to holistically comprehend the effect of biopesticides against vascular plant fungi on the environment and/or on the target plant holobiont have not yet been conducted. One of the few works available in the literature on this topic was conducted by Kotula et al. [238], who compared two ML techniques (random forest and k-nearest neighbour) to predict direct and indirect non-target impacts of species in parasitoid–host networks, in order to use these tools in a biological control framework.

### 6.5. Simulating Plant–Pathogen–Biocontrol Interactions

AI, boosted through deep learning, can simulate the complex interactions between plants/holobionts, pathogens and BCAs, being a powerful tool to manage these complex datasets. Such models enable the prediction of pathogen behaviour under various environmental conditions, as well as the efficacy of BCAs. This approach may therefore help to refine biological control strategies based on different scenarios and conditions [239]. Until now, however, the vast majority of studies unravelling plant–microbial interactions using AI have been focused on host–pathogen interactions. Thus, Sperschneider [240] has highlighted future opportunities for ML as a tool for disentangling plant–pathogen interactions using high-throughput data. Interestingly enough, Wang and Zou [111] conducted a deep learning meta-analysis to train, evaluate, and interpret the soil microbiome, facilitating the exploration of underlying microbial indicators distinguishing between diseased and healthy soils in the particular case of Fusarium and Verticillium wilts. Notwithstanding, AI models could also simulate the impact of changing soil moisture or temperature on the interaction between a beneficial *Trichoderma* strain, a vascular pathogen, and the host. Indeed, although not being an example of vascular fungi, a non-linear ML based on the Artificial Neural Network technique has already been successfully applied to assess the biocontrol efficacy of *Trichoderma* spp. in baby leaf plants against soil-borne fungal pathogens [241].

Another promising tool for studying and simulating the interactions occurring in the plant holobiont is digital twin technology, which is expected to transform agriculture. A digital twin “is a digital equivalent of a real-life object of which it mirrors its behaviour and its states over its lifetime in a virtual space” [242,243,244]. Digital twin technology can provide a real-time representation of agricultural ecosystems, utilizing high-fidelity modelling to simulate the complex interactions between pathogens, soil, plants, and their associated microbiomes, that is to say, those taking place between plant holobionts and the surrounding environment. By integrating automation and advanced AI models, digital twins would enable the prediction and monitoring of disease outbreaks, and they may provide decision support for the optimal inoculation of BCAs. Through continuous simulation and optimization of environmental and biological conditions, this revolutionary tool may virtually predict how a vascular fungal disease will develop and respond with timely (biocontrol) interventions.

## 7. Genome Editing, RNA Interference, and Functional Peptides: Innovative, High-Potential Technologies Yet to Be Fully Implemented in Biocontrol

### 7.1. Genome Editing Technologies

Genome editing (GE) consists of the modification (deletions, insertions, and replacements) of genomic DNA at specific target sites in a wide range of cell types and organisms. The final goals are the acquisition of novel genetic traits, inactivation of target genes, or correction of gene mutations [245]. Zinc finger nucleases (ZFNs), transcription activator-like effector nucleases (TALENs), and the RNA-guided CRISPR (Clustered Regularly Interspaced Short Palindromic Repeats)-Cas (CRISPR-associated) nucleases systems are the three primary GE approaches, the latter being the most commonly used due to its simple design, low cost, great efficiency, remarkable repeatability, and quick cycle [246,247,248].

GE has revolutionized plant disease resistance breeding by providing sustainable solutions to combat pathogens, thereby contributing to global food security. Additionally, GE allows enhancing yield and tolerance to abiotic stresses [249,250]. GE, and, specifically, CRISPR–Cas is also implemented to comprehend plant–microbe interactions, including the role of endophytic microorganisms (and their bioactive compounds) that can potentially be used as BCAs or biocontrol tools [251].

CRISPR/SpCas9-mediated (DNA-cutting enzyme derived from *Streptococcus pyogenes* that, guided by RNA, enables precise gene editing by creating double-strand breaks at specific DNA sequences) technology has been widely utilized in different fungal species. Regarding vascular fungi, this tool has proven effective to edit the genome of *F. oxysporum* [139,143,144,252] (Table 1). Thus, CRISPR-based approaches have been successfully used to investigate and alter genes involved in pathogenicity, colonization, and fungicide resistance, providing a deeper understanding of the molecular mechanisms of these processes, as well as exploring new disease control strategies (e.g., [139,253]). An interesting alternative to easily obtain new genotypes capable of acting as BCAs is the induction of mutations in virulent genotypes. This strategy could convert virulent pathogens into “disarmed” variants, which could then compete with their pathogenic counterparts or trigger plant defence responses [254]. By “transforming” harmful pathogens into protective BCAs, this technology offers great and novel biotechnological potential within disease management frameworks, thereby contributing to reduce the need for chemical fungicide inputs.

The biocontrol activity of beneficial microbes against phytopathogenic fungi could be improved using GE approaches aimed to modify specific metabolic pathways, thereby triggering the biosynthesis of antibiotics, cell wall-degrading enzymes, secreted proteins and secondary compounds that are synthesized either at low or null levels in wild-type strains, or to identify new microbial bioactive compounds [255]. This would generate more effective biocontrol strains to be released under field conditions, avoiding the introduction of transgenes into the environment [256]. The CRISPR/Cas9 system has also been successfully implemented to generate endophytic mutants with increased bioactive compound production [255,257] and enhanced enzymatic expression [258,259]. Finally, this GE system has also provided relevant information regarding the colonization process, the role in plant growth promotion, and improved plant pathogen tolerance mediated by beneficial bacteria, including those displaying endophytic lifestyles [251]. For instance, this GE system was implemented to study the molecular processes involved in the biocontrol and plant growth-promoting properties of *Bacillus subtilis* HS3 and *B. mycoides* EC18 [260].

Moreover, GE offers potential applications in tailoring microbial communities to achieve the desired outcomes in plants. This can be achieved by engineering specific genetic traits in microbial strains to enhance their beneficial effects on plant growth, nutrient uptake, and disease resistance [261]. However, manipulating microbiomes in agroecosystems faces important obstacles to overcome due to the complexities (e.g., agronomic practices, climatic conditions, pedological characteristics, etc.) found in farm environments. Some authors have proposed GE of the host plant as another approach through which microbiomes could be manipulated [262]. By targeting key genes or traits involved in recruiting and controlling specific microbiomes, the ability of the host to shape its microbial community could be enhanced, leading to improved disease resistance and plant health. This approach aligns with our own findings [159], in which the genotype of olive cultivars seems to be crucial not only for the recruitment of beneficial microbes assisting in the suppression of *V. dahliae* but also in shaping the indigenous microbiota inhabiting the roots of olive varieties tolerant to this pathogen. Identifying the host genetic factors and keystone microorganisms involved in the tolerance/resistance phenotype (holobiont context) could thus pave the way for more effective microbiome-based disease management strategies.

Prime editing (PE) is another, more advanced, GE technique that was developed by Anzalone et al. [263] to improve the traditional CRISPR/Cas9 system. PE allows more precise changes in the DNA and does not require double-strand breaks, thereby reducing the risk of errors and unwanted damage [264,265,266]. So far, PE has been successfully applied in a variety of plant and animal cells, as well as in the model microorganism *Escherichia coli*. It has also shown potential in breeding and genomic functional studies of animals and plants, disease treatment, and modification of microbial strains [264] (and references therein). Thus, PE could also be implemented in the field of plant–microbe interactions, enhancing the capabilities of beneficial and/or symbiotic microorganisms [267]. PE may facilitate, for instance, the identification of microbial candidate genes controlling beneficial traits. In this sense, PE will be very useful in the design of beneficial microbial communities to improve crop productivity since direct links between desirable agronomic characteristics and microbial genes can be established. In summary, GE techniques have enormous potential in combating crop diseases. Yet, potential drawbacks must be overcome, such as the possibility of mistargeting and causing unexpected side effects [268].

### 7.2. RNA Interference

RNA interference (RNAi), present in most eukaryotic cells, is a natural mechanism capable of suppressing the expression of specific genes, degrading messenger RNA (mRNA) and/or inhibiting translation, i.e., post-transcriptional regulation. This process, known as ribointerference, is part of an evolutionarily conserved immune system designed to protect cells from incoming foreign DNA [269]. RNAi runs through small non-coding RNAs, specifically small interfering RNAs (siRNAs) and microRNAs (miRNAs), which pilot ribonucleoprotein complexes named RNA-induced silencing complexes (RISCs) responsible for targeting specific RNA sequences for silencing. This targeting occurs when double-stranded RNAs (dsRNAs) enter and are processed into siRNAs by plant cells. These small molecules are then bound to complementary RNA sequences, originating their degradation or preventing their translation [270]. RNAi has risen as a puissant and useful tool in the control of diseases and pests relevant in agriculture, particularly due to the phenomenon known as cross-kingdom RNAi, by which plants and their pathogens can exchange siRNAs to regulate gene expression [271]. The overriding RNAi approaches enforced in crop protection are spray-induced gene silencing (SIGS), followed by host-induced gene silencing (HIGS), virus-induced gene silencing (VIGS), and microbe-induced gene silencing (MIGS) [98,272,273]. In the SIGS system, exogenous sequence-specific dsRNAs/siRNAs are directly sprayed onto the host plant to control diseases [274]. These RNAs can be taken up by plant cells and processed into siRNAs or absorbed by fungi from the plant surface and transformed into siRNAs by fungal cells [275,276]. Therefore, SIGS is a time-efficient and genetically modified organism (GMO)-free method that can be applied more flexibly to address emerging phytopathological challenges within a reasonable timeframe. HIGS is a transgene-mediated technique [277]. The dsRNAs are synthetised from engineered plant genomes and subsequently processed into siRNAs. During pathogen infection, siRNAs can be transported from a plant cell into the pathogen cell and specifically silencing a fungal target gene [276,278,279]. HIGS shows as highly efficient to enhance plant resistance by simultaneously silencing one or more fungal genes in an environmentally friendly way. Nonetheless, the generation of transgenic plants is challenging and time-consuming and requires the public acceptance of GMOs. VIGS technology employs RNA viruses/mycovirus to carry target gene segments and induce the silencing of endogenous genes through viral replication and transcription in plant/fungus. It is prominently used in plant gene function studies [280,281,282]. VIGS allows the horizontal transmission of the mentioned gene segments from hypovirulent to virulent strains, generating dsRNAs/siRNAs to protect plants in the field [283]. In the MIGS technique, siRNAs are produced in a donor microorganism and then transferred to a recipient pathogen silencing the target gene. This approach provides plant protection against pathogens by using a GMO able to continuously supply siRNAs. However, this strategy requires thorough risk assessments and additional fundamental research to fully explore its potential.

Delivery strategies of dsRNA for diseases management can be broadly divided into transformative (endogenous delivery) and non-transformative (exogenous delivery). Transformative strategies involve the introduction of genes encoding fungicidal dsRNA into plants by genetic engineering, resulting in the endogenous production of dsRNA within the crop plant. Non-transformative strategies entail a broader range of delivery methods, although all of them rely on the topical application of dsRNA synthesized externally from the target crop plant, either produced in vitro or through microorganisms [284]. The use of microorganisms as biofactories to produce dsRNA in vivo is a promising avenue for enhancing biocontrol. dsRNA synthesized in microorganisms can be extracted for direct application, delivered within dead microbial cells, or applied through living microorganisms (bacterium-mediated RNAi) [285]. As an advantage, there is no need to transform the plant, although transformation of the microbial biofactory to produce the dsRNAs is still required. In this approach, genetically modified microorganisms, such as bacteria (e.g., *Pseudomonas* spp., *Echerichia coli*) and mycorrhizal fungi, can be engineered to express and deliver siRNAs in vivo, directly in the plant rhizosphere or within plant tissues. So far, to our knowledge, this approach has not been used to confront vascular fungi. However, the RNaseIII-null mutant strain of *Escherichia coli* HT115(DE3) was used to produce biologically active dsRNA against genes involved in aflatoxin production in *Aspergillus flavus (AflC)* or virulence in *Botrytis cinerea (BcSAS1)* [286]. Similarly, a MIGS strategy using *T. harzianum* has been recently developed. This fungus was engineered to produce dsRNA targeting essential genes of *V. dahliae* and *F. oxysporum* [98]. By using this approach, significant growth inhibition of these pathogens, leading to enhanced protection in both dicotyledonous (cotton) and monocotyledonous (rice) plants, was demonstrated. Some of these microorganisms can colonize plant roots and act as natural platforms for the continuous production and release of siRNAs, which are then absorbed by the plant and/or pathogen cells. Once inside the target pathogen, the siRNAs trigger gene silencing, reducing the pathogen’s ability to infect or damage the plant. This strategy offers a biological, sustainable alternative to traditional chemical treatments, as the microorganisms act as natural carriers of siRNAs, potentially reducing the need for synthetic chemicals or genetically modified plants. The use of microorganism-produced RNAi has the added benefit of leveraging the plant’s own defence mechanisms, as siRNAs produced in situ can participate in cross-kingdom RNAi, thereby enhancing the plant’s ability to fend off pathogens [287,288].

Some examples of *V. dahliae* [98,148] and *F. oxysporum* genes targeted through SIGS, HIGS, VIGS, and MIGS are summarized in Table 1 and Table 2. Even though all these ‘IGS’ techniques have successfully conferred RNAi-mediated resistance against several vascular fungal pathogens [283,289], thorough field research is needed to untangle their full potential in crop protection. Moreover, ensuring the efficiency and stability of siRNAs delivered by microorganisms still constitutes a challenge.

#### RNAi and Nanoparticles: The Synergy of Two Groundbreaking Technologies

As mentioned in Section 5.2, NPs are excellent delivery vehicles and may help improve the durability and uptake of RNAi due to their properties. Their small size provides a high surface area/volume ratio for effective cargo binding. Additionally, many NPs are small enough to cross the plant cell wall and membrane barriers, being able to act efficiently against a given pathogen without producing side effects out of the selected target. On the other hand, ‘IGS’ technologies are limited by the unstable nature of dsRNAs that are applied topically, which makes them very labile to environmental conditions. In order to achieve greater RNAi effectiveness, fungal-specific dsRNAs must be protected from degradation by RNases to prolong their activity. NPs can function as cost-effective and eco-friendly dsRNA carriers to improve the efficacy of the SIGS approach [289], making the microbial production of RNAi a more robust and scalable solution for controlling plant pathogens, including vascular fungi [290,291]. By optimizing both delivery mechanisms and formulation processes, the use of RNAi-producing microorganisms could become a key component in future biological control strategies. NCs have been used for nucleic acid delivery in plant biotechnology since 1980s [292,293], carbon nanotubes being among the first non-cytotoxic NPs capable to cross the plant cell wall and membrane [294]. Mitter et al. [295] showed that dsRNAs targeting pathogen genes could be delivered into intact plant cells through the topical spraying of layered double hydroxide (LDH) NPs to load them onto dsRNA. They demonstrated that LDH NP-loaded dsRNA simultaneously protected and released dsRNA in a controlled way and provided longer-term protection against two plant viruses than naked dsRNA.

Some NCs have already been showed to trigger RNAi in phytopathogens through SIGS application of dsRNA-NC composites [295]. For instance, dsRNA-LDH nanosheets targeting three essential genes (*CYP51*, *Chs1*, and the elongation factor 2 (*EF2*)) in *F. oxysporum* and using different methods provided protection against the pathogen [140]. However, biosafety assays of these NCs are still needed to discard certain risks, since LDH aggregates are toxic to mice [296] and green algae [297]. Other NCs have shown to elicit the RNAi of plant genes through SIGS application of dsRNA/siRNA-NC composites, and they could thus be potentially used for carrying RNAi and controlling phytopatogenic fungi. Among them, DNA nanostructures, carbon dots, single-walled carbon nanotubes (SWNTs), gold nanoclusters, and cell-penetrating peptides stand out. Nevertheless, several factors such as target gene selection, dsRNA/siRNA design parameters, dsRNA uptake mechanisms, barriers to dsRNA uptake delivery strategies, application methods, dsRNA doses and sizes, and environmental stability can determine the efficacy of this tool to trigger successful RNAi in plant–fungal interactions [289,298,299,300,301,302]. NC size has been a major factor in figuring out whether dsRNAs/siRNAs are able to penetrate into plant cells. To cross the plant cell wall and membrane, which usually have a size exclusion limit of < 20 nm, ultra-small NPs such as SWNTs, carbon dots, and DNA nanostructures with large loading dsRNA/siRNA abilities have been engineered. Nonetheless, it has been shown that large NCs such as LDH and CNPs loaded with RNAi conferred protection against phytopathogens/pests, indicating that gene silencing can occur without NP internalization or by the internalization of the endosome pathway.

The use of NCs allows the dsRNA dosage to be reduced, which diminishes production costs and the frequency of field application. Thus, ‘IGS’ combined with NCs has the potential to provide a more efficient, economical, and sustainable solution for the control of crop diseases caused by phytopathogenic vascular fungi.

**Table 2:** Examples of genes silenced in fungal vascular pathogens by RNAi approaches *.

Fungal Pathogen	Host	RNAi Approach	RNA	Target Gene (s)	Role of Target Gene (s)	Silencing Outcome	Reference
*F. oxysporum*	Soybean	HIGH	hpRNA	*CYP51B*	Target for azole fungicide, hyphal growth	Enhanced plant resistance	[99]
		MIGS (solid culture)	hpRNA	*FoPMT2*	Fungicide target against phytopathogens	Inhibited mycelial growth	[98]
	Rice	MIGS	hpRNA	*FoPMT2*	Fungicide target against phytopathogens	Reduced disease development	[98]
*F. oxysporum* f. sp. *cubense*		Liquid culture	sdRNA	14 genes	Conidia germination	Inhibited conidia germination	[112]
		Liquid culture	siRNA	*VEL, FTF1*	Regulator of (a)sexual deveploment, secondary metabolims and virulence	Reduced mycelial growth, reduced conidiophore count	[278]
	Banana	HIGS	hpRNA	*VEL, FTF1*	Regulator of (a)sexual deveploment, secondary metabolims and virulence	Enhanced plant resistance	[278]
		Tranformants	hpRNA	*SEG1*	Pathogen parasitic growth	Reduced conidiophore count, reduced fusarium wilt virulence	[113]
		Liquid culture	dsRNA	*ERG6, ERG11*	Ergosterol biosynthesis	Inhibited fungicide tolerance	[303]
	Banana	HIGS	hpRNA	*ERG6, ERG11*	Ergosterol biosynthesis	Enhanced plant resistance	[303]
*F. oxysporum* f. sp. *conglutigans*	Arabidopsis	HIGS	hpRNA	*FRP1, OPR, FOW2*	Pathogenicity and (*FRP1* and *FOW2*) jamonic acid synthesis (*OPR*)	Enhanced plant resistance	[142]
*F. oxysporum* f. sp. *lycopersici*		Transformants	hpRNA	*FMK1, HOG1, PBS2*	MAP Kinase signalling genes	Altered conidal morphology, reduced virulence on tomato	[124]
		Transformants	hpRNA	*FOW2*	Pathogenicity	Mycelial growht defects, reduced conidia production, reduced virulence on tomato	[100]
	Tomato/ Arabidopsis	HIGS	hpRNA	*FOW2*	Pathogenicity	Enhanced plant resistance	[125]
	Tomato	HIGS	hpRNA	*CHSV*	Chitin synthesis	Enhanced plant resistance	[125]
		Transformants	hpRNA	*PEX6*	Peroxisomal biogenesis	Mycelial growht defects, reduced conidia production, reduced virulence on tomato	[304]
	Tomato	HIGS	hpRNA	*ODC*	Fungal growth	Enhanced plant resistance	[126]
	Tomato	HIGS	hpRNA	*PEX6*	Peroxisomal biogenesis	Enhanced plant resistance	[127]
	Tomato	HIGS	hpRNA	*GAS1*	Fungal cell wall biosynthesis and morphogenesis	Enhanced plant resistance	[127]
		Transformants	hpRNA	*FoFLP1, FoFLP3, FoFLP4, FoFLP5*	Cell adherence	Reduced conidia production, reduced virulence on tomato	[128]
		Liquid culture	dsRNA	*FolRDR1*	Pathogen development	Reduced conidia production	[129]
	Tomato seedlings	SIGS	dsRNA	*FolRDR1*	Pathogen development	Inhibited disease development	[129]
	Tomato	HIGS	hpRNA	*FoFLP1, FoFLP4, FoFLP5*	Cell adherence	Enhanced plant resistance	[305]
*F. oxysporum* f. sp. *radicis-lycopersici*		Transformants	hpRNA	*CYP51, CHS1, EF2*	Pathogenesis, chitin synthesis and ribosomal translocation.	Lower virulence on tomato	[140]
		Liquid culture	hpRNA	*CYP51, CHS1, EF2*		Inhibited mycelial growth	[140]
	Tomato seedling	SIGS	dsRNA	*CYP51, CHS1, EF2*		Inhibited disease development	[140]
*Verticillium dahliae*	Arabidopsis	HIGS	dsRNA/ sRNA	*DCL*	Vesicle trafficking	Enhanced plant resistance	[276]
	Arabidopsis/ Tomato	HIGS	dsRNA	*Ave1, Sge1 and NLP1*	Pathogenicity factors	Enhanced plant resistance	[306]
	Cotton	HIGS	dsRNA	*VdRGS1*	Regulator of G protein involved in spore production, hyphal development and microsclerotia formation	Enhanced plant resistance	[148]
	Cotton	HIGS	ds RNA	*VdILV2 and VdILV6*	Branched-chain amin oacid synthesis	Drastic reduction in disease development	[307]
	*Nicothiana benthamiana*/*Arabidopsis thaliana*	HIGS	dsRNA	*VdAK*	Fungal metabolism, conidiation, and pathogenicity	Enhanced plant resistance	[308]
	Arabidopsis	SIGS	dsRNA	*Vd-DCL1/2 Vd-DCTN1 VdSAC1*	Vesicle trafficking	Reduced disease symptoms and fungal biomass (55% with DCL1 + DCL2-, 60% with DCTN1 + SAC1)	[309]
	Cotton	HIGS	dsRNA/ siRNA	*VdH1*	Melanized microsclerotia formation	50–70% reduced disease symptoms	[310]
	Cotton/Rice	MIGS	dsRNA/ siRNA	*VdPMT2*	Fungicide target against phytopathogens	Inhibited fungal growth	[98]
	Cotton	HIGS	dsRNA	*VdThit*	Pathogenesis	Enhanced plant resistance	[111]

* Based on information compiled by Ray et al. [289] and Liu et al. [283]. For gene nomenclature, consult these two references and the references included in this table.

### 7.3. Functional Peptides

Functional peptides are short sequences of amino acids (50–60) displaying biological activities beyond their nutritional value. They originate either from natural sources (microorganisms, animals, or plants) or through artificial synthesis [311,312]. Among their properties, the ability to interact with a range of biological targets (e.g., receptors, membranes, or enzymes), which can then trigger antibacterial, antiviral, or antifungal activities, can be highlighted [312,313,314]. Additionally, effectiveness against insects and weeds as well as the ability to induce resistance and plant growth promotion have been reported [311,315]. Functional peptides in plant disease management offer diverse advantages, including abundant raw material sources, high activity, and environmental safety [315]. There are different types of functional peptides. Some of the best known are the so-called antimicrobial peptides (AMP) that exert their effects through different mechanisms of action which have been recently and thoroughly reviewed [312].

Some bacterial-derived AMPs such as cyclic lipopeptides (e.g., iturin and surfactin) and bacteriocins (e.g., amylocyclicin and ericin S) produced by *Bacillus* spp., and cyclodepsipeptides synthetized by *Pseudomonas* spp. trigger effective defence responses in plants attacked by bacterial and fungal pathogens [312]. Surfactin A, purified from *Bacillus subtilis* NH-100 and *Bacillus* sp. NH-217, has strong antifungal activity against *F. oxysporum* [103]. Similarly, fungal-derived AMPs have been tested against important pathogens of extensively grown crops. A good example is Trichokonin VI, a peptaibol [316] produced by *Trichoderma pseudokoningii* and able to control *F. oxysporum* by inducing metacaspase-independent apoptotic cell death in this fungal pathogen [104]. Plants are another source of AMPs [317,318,319], defensins being the largest family of this type of peptides expressed in this kingdom [311,320,321,322,323,324,325,326,327]. For instance, floral defensins from petunia showed potent growth inhibition of pathogenic filamentous fungi, especially towards *F. oxysporum* in vitro [105]. High-level constitutive expression of petunia defensin in banana plants led to significant resistance against *F. oxysporum* f. sp. *cubense* in both in vitro and ex vivo bioassays [116]. In addition, plants are able to synthetize other AMPs like knottin-like peptides, thionins [328], snakins [329], hevein-like peptides, and lipid transfer proteins [330]. Thus, Muramoto et al. [155] demonstrated that purothionin from wheat endosperm showed strong toxicity to *C. fimbriata* in vitro. Moreover, they showed that transgenic sweet potato expressing the AMP α-hordothionin from barley was a promising way to reduce losses due to black rot caused by *C. fimbriata*. Sweany et al. [331] demonstrated the antifungal activity of the two synthetic functional peptides GV185 and GV187 against *F. oxysporum* f. sp. *vasinfectum* and *V. dahliae*, among other fungal pathogens. For more information on the use of functional peptides for the control of phytopathogenic vascular fungi, see Table 1.

It is worth mentioning that the antifungal activity of functional peptides may be reduced in plant tissues or within the vascular system [332] since they can be degraded by proteases [333]. Certainly, these problems can be mitigated or overcome by modification of the natural peptides or through different formulations [325] (e.g., nanoencapsulation; see Section 5.2), although these strategies increase the complexity of peptide development and production [312]. Furthermore, the usage of functional peptides in the biocontrol of vascular fungal diseases faces some other challenges. Special attention is being currently paid to the design of peptides displaying various mechanisms of action, as they could effectively counteract potential pathogen resistance and enhance different aspects of plant physiology. Likewise, small amounts (e.g., milligrams) of peptides are required for in vitro screening in laboratories. However, assays at the plant or field scales would require moderate-to-large quantities (e.g., grams). The future of functional peptides as plant protection products may help in reducing the use of chemicals in agriculture, although their success will certainly depend on the capacity to produce high quantities using industrial platforms.

## 8. Concluding Remarks

**Figure 3:**
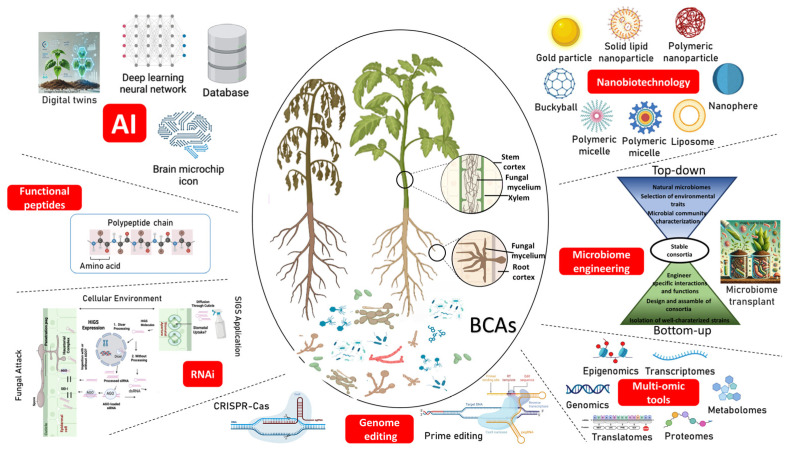
A graphical overview of the cutting-edge technologies mentioned in this review and aimed at improving biocontrol strategies for vascular fungal diseases. The figure was created using icons and templates from the free online BioRender (https://www.biorender.com/, accessed on 12 December 2024), except for the digital twins and microbiome transplant images, which were generated with the free online version of ChatGPT (https://chatgpt.com/, accessed on 12 December 2024). The acronyms used are defined as follows: biological control agent (BCA), artificial intelligence (AI), and RNA interference (RNAi).

Future agriculture will be driven by global challenges that demand urgent actions, particularly the need to (i) make agroecosystems more sustainable, (ii) mitigate (or/and adapt to) the impacts of climate change, and (iii) preserve the genetic diversity of cultivated plants. One of the core issues in this scenario is the improvement of strategies to achieve better control of plant diseases, including those caused by vascular fungi, which pose significant challenges due to their own biology. The traditional inconsistency associated with biocontrol strategies must be carefully addressed, focusing on solutions that contribute to soil biodiversity preservation, including the microbiome, which is essential to keep healthy agroecosystems. The immoderate, and frequently inadequate, use of agrochemicals has led to undesirable consequences for living organisms and the environment, underscoring the urgent need to adopt more sustainable practices. To accomplish the objectives of the European Green Deal in general, and with the effective control of fungal vascular diseases in particular, will be difficult unless viable and sustainable alternatives are developed soon. In this framework, Agriculture 4.0—a result of the fourth industrial revolution—emerges as a life-changing force. This new agriculture model integrates sophisticated digital technologies such as AI, ML, big data, the Internet of Things (IoT), nanotechnology, digital twins, robotics, and automation to revolutionize forefront farming. These innovations enable real-time monitoring and adaptive responses to environmental and crop health changes, including precision in biocontrol strategies. Agriculture 4.0 enables the optimization of the use of BCAs, significantly minimizing reliance on chemical inputs and improving the precision and efficiency of crop management. This approach fits perfectly with the One Health concept, which focuses on the links between human, animal, and environmental health and stresses the need for a holistic vision to achieve agricultural sustainability. The transition to smarter, more automated, and connected agricultural systems is essential to reduce greenhouse gas emissions, a major aspect as the agri-food sector accounts for approximately 26% of global CO₂ emissions. By leveraging these advanced technologies (Figure 3), agriculture can not only increase productivity but also reduce its environmental impact, conserve biodiversity, and ensure a more sustainable future for both ecosystems and human health. Certainly, novel and more effective biocontrol frameworks, assisted and improved by these cutting-edge technologies, are called to play a relevant role to reach these goals. As mentioned in this review, the groundbreaking technologies compiled and discussed here still need to overcome difficulties posed by the particular case of fungal vascular pathogens. The near future will tell which one(s) could be more relevant and/or successful in confronting them, but examples are already available in the recent literature, as shown in Table 1 and Table 2, offering a glimpse into potential future directions.

## Figures and Tables

**Table 1:** Summary of technologies mentioned in this review and [studies] implementing them in the biocontrol of fungal vascular pathogens (plant hosts are indicated when known). See main text for details.

Pathogens/Host	Genome Editing	Microbiome Engineering	RNAi	Funtional Peptides	Nanobiotechnology	Artificial Intelligence
*Fusarium oxysporum*			[98,99,100,101,102]	[103,104,105]	[106,107,108,109]	[110,111]
*F. oxysporum* f. sp. *cubense*/banana			[112,113,114,115]	[116]	[117]	[118]
*F. oxysporum* f. sp. *ciceris*/chickpea						[119,120,121]
*F. oxysporum* f. sp. *cucumerinum*/cucumber					[117,122]	[123]
*F. oxysporum* f. sp. *lycopersici*/tomato			[124,125,126,127,128,129,130,131]		[117,132,133,134]	[135]
*F. oxysporum* f. sp. *phaseoli/*bean		[97]			[136]	[137]
*F. oxysporum* f. sp. *fragrariae/*strawberry						[138]
*F. oxysporum* f. sp. *radicis lycopersici*/tomato	[139]		[140]			[141]
*F. oxysporum* f. sp. *conglutinans*/cabbage			[142]			
*F. oxysporum* f. sp. *vasinfectum*/cotton	[143,144]		[145]		[146]	
*F. oxysporum* f. sp. *melonis*/melon						[123]
*F. oxysporum* f. sp. *lactucae*/lettuce						[147]
*Verticillium dahliae*/cotton/potato/olive			[98,148]		[149]	[111,150,151,152,153,154]
*Verticillum albo-atrum*/potato						[151]
*Ceratocystis fimbriata/*eucaliptus/potato				[155]		[156]
*Ophiostoma ulmi*/elm						[157]
*Ophiostoma novo-ulmi*/elm						[157]

## Data Availability

Data sharing not applicable.

## References

[1] United Nations (2019). World Population Prospects 2019: Highlights.

[2] Savary S., Willocquet L., Pethybridge S.J., Esker P., McRoberts N., Nelson A. (2019). The global burden of pathogens and pests on major food crops. Nat. Ecol. Evol..

[3] Rizzo D.M., Lichtveld M., Mazet J.A.K., Togami E., Miller S.A. (2021). Plant health and its effects on food safety and security in a One Health framework: Four case studies. One Health Outlook.

[4] FAO (2017). The Future of Food and Agriculture: Trends and Challenges.

[5] Damalas C.A., Eleftherohorinos I.G. (2011). Pesticide exposure, safety issues, and risk assessment indicators. Int. J. Environ. Res. Public Health.

[6] Rani L., Thapa K., Kanojia N., Sharma N., Singh S., Grewal A.S., Srivastav A.L., Kaushal J. (2021). An extensive review on the consequences of chemical pesticides on human health and environment. J. Clean. Prod..

[7] Foong S.Y., Ma N.L., Lam S.S., Peng W., Low F., Lee B.H., Alstrup A.K.O., Sonne C. (2020). A recent global review of hazardous chlorpyrifos pesticide in fruit and vegetables: Prevalence, remediation and actions needed. J. Hazard. Mater..

[8] Bebber D.P., Ramotowski M.A., Gurr S.J. (2013). Crop pests and pathogens move polewards in a warming world. Nat. Clim. Change..

[9] Chaloner T.M., Gurr S.J., Bebber D.P. (2021). Plant pathogen infection risk tracks global crop yields under climate change. Nat. Clim. Chang..

[10] Fisher M.C., Henk D.A., Briggs C.J., Brownstein J.S., Madoff L.C., McCraw S.L., Gurr S.J. (2012). Emerging fungal threats to animal, plant and ecosystem health. Nature.

[11] Panzavolta T., Bracalini M., Benigno A., Moricca S. (2021). Alien invasive pathogens and pests harming trees, forests, and plantations: Pathways, global consequences and management. Forests.

[12] McDonald B.A., Stukenbrock E.H. (2016). Rapid emergence of pathogens in agro-ecosystems: Global threats to agricultural sustainability and food security. Philos. Trans. R. Soc. B..

[13] Baedke J., Fábregas-Tejeda A., Delgado A.N. (2020). The holobiont concept before Margulis. J. Exp. Zool. Part B Mol. Dev. Evol..

[14] Lyu D., Zajonc J., Pagé A., Tanney C.A.S., Shah A., Monjezi N., Msimbira L.A., Antar M., Nazari M., Backer R. (2021). Plant holobiont hteory: The phytomicrobiome plays a central role in evolution and success. Microorganisms.

[15] Mesny F., Hacquard S., Thomma B.P. (2023). Co-evolution within the plant holobiont drives host performance. EMBO Rep..

[16] Garrett K.A., Dendy S.P., Frank E.E., Rouse M.N., Travers S.E. (2006). Climate change effects on plant disease: Genomes to ecosystems. Annu. Rev. Phytopathol..

[17] Fisher M.C., Hawkins N.J., Sanglard D., Gurr S.J. (2018). Worldwide emergence of resistance to antifungal drugs challenges human health and food security. Science.

[18] Mitra D., Singh K.P., Jahagirdar S., Sarma B.K. (2021). Emerging plant diseases: Research status and challenges. Emerging Trends in Plant Pathology.

[19] Zhai Z., Martínez J.F., Beltran V., Martínez N.L. (2020). Decision support systems for agriculture 4.0: Survey and challenges. Comput. Electron. Agric..

[20] Ma M., Taylor P.W.J., Chen D., Vaghefi N., He J.-Z. (2023). Major soilborne pathogens of field processing tomatoes and management strategies. Microorganisms.

[21] Thangavelu R., Loganathan M., Arthee R., Prabakaran M., Uma S. (2020). Fusarium wilt: A threat to banana cultivation and its management. CABI Rev..

[22] Jacobi W.R., Koski R.D., Negron J.F. (2013). Dutch elm disease pathogen transmission by the banded elm bark beetle Scolytus. Forest Pathology.

[23] Hughes M.A., Smith J.A., Ploetz R.C., Kendra P.E., Mayfield A.E., Hanula J.L., Hulcr J., Stelinski L.L., Cameron S., Riggins J.J. (2015). Recovery plan for laurel wilt on redbay and other forest species caused by *Raffaelea lauricola* and disseminated by *Xyleborus glabratus*. Plant Health Prog..

[24] Yadeta K.A., Thomma B.P.H.J. (2013). The xylem as battleground for plant hosts and vascular wilt pathogens. Front. Plant Sci..

[25] Martín J.A., Fuentes-Utrilla P., Gil L., Witzell J. (2010). Ecological factors in Dutch elm disease complex in Europe—A review. Ecol. Bul..

[26] Harrington T.C., Gonthier P., Nicolotti G. (2013). Ceratocystis diseases. Infectious Forest Diseases.

[27] Keykhasaber M., Thomma B.P.H.J., Hiemstra J.A. (2018). Verticillium wilt caused by *Verticillium dahliae* in woody plants with emphasis on olive and shade trees. Eur. J. Plant Pathol..

[28] Bubici G., Kaushal M., Prigigallo M.I., Gómez-Lama Cabanás C., Mercado-Blanco J. (2019). Biological control agents against Fusarium Wilt of banana. Front. Microbiol..

[29] Montes-Osuna N., Mercado-Blanco J. (2020). Verticillium wilt of olive and its control: What did we learn during the last decade?. Plants.

[30] Bernier L., Asiegbu F., Kovalchuk A. (2022). Dutch elm disease. Forest Microbiology.

[31] López-Escudero F.J., Mercado-Blanco J. (2011). Verticillium wilt of olive: A case study to implement an integrated strategy to control a soil-borne pathogen. Plant Soil.

[32] Dita M., Barquero M., Heck D., Mizubuti E.S.G., Staver C.P. (2018). Fusarium wilt of banana: Current knowledge on epidemiology and research needs toward sustainable disease management. Front. Plant Sci..

[33] Scheffer R.J., Strobel G.A., Mukerji K.G., Garg K.L. (2020). Dutch elm disease, a model tree disease for biological control. Biocontrol of Plant Diseases.

[34] Bahadur A., Mirmajlessi S.M. (2021). Current status of *Fusarium* and their management strategies. Fusarium—An Overview of the Genus.

[35] Kowalska B. (2021). Management of the soil-borne fungal pathogen—*Verticillium dahliae* Kleb. causing vascular wilt diseases. J. Plant Pathol..

[36] Harman G., Howell C., Viterbo A., Chet I., Lorito M. (2004). *Trichoderma* species—Opportunistic, avirulent plant symbionts. Nat. Rev. Microbiol..

[37] Shoresh M., Harman G.E., Mastouri F. (2010). Induced systemic resistance and plant responses to fungal biocontrol agents. Annu. Rev. Phytopathol..

[38] Maldonado-González M.M., Bakker P.A., Prieto P., Mercado-Blanco J. (2015). *Arabidopsis thaliana* as a tool to identify traits involved in *Verticillium dahliae* biocontrol by the olive root endophyte *Pseudomonas fluorescens* PICF7. Front. Microbiol..

[39] Markakis E.A., Tjamos S.E., Antoniou P.P., Paplomatas E.J., Tjamos E.C. (2016). Biological control of Verticillium wilt of olive by *Paenibacillus alvei*, strain K165. BioControl.

[40] Shafi J., Tian H., Ji M. (2017). *Bacillus* species as versatile weapons for plant pathogens: A review. Biotechnol. Biotechnol. Equip..

[41] Gómez-Lama Cabanás C., Legarda G., Ruano-Rosa D., Pizarro-Tobías P., Valverde-Corredor A., Niqui J.L., Triviño J.C., Roca A., Mercado-Blanco J. (2018). Indigenous *Pseudomonas* spp. strains from the olive (*Olea europaea* L.) rhizosphere as effective biocontrol agents against *Verticillium dahliae*: From the host roots to the bacterial genomes. Front. Microbiol..

[42] Gómez-Lama Cabanás C., Ruano-Rosa D., Legarda G., Pizarro-Tobías P., Valverde-Corredor A., Triviño J.C., Roca A., Mercado-Blanco J. (2018). Bacillales members from the olive rhizosphere are effective biological control agents against the defoliating pathotype of *Verticillium dahliae*. Agriculture.

[43] Castro D., Torres M., Sampedro I., Martínez-Checa F., Torres B., Béjar V. (2020). Biological control of Verticillium Wilt on olive trees by the salt-tolerant strain *Bacillus velezensis* XT1. Microorganisms.

[44] Gómez-Lama Cabanás C., Fernández-González A.J., Cardoni M., Valverde-Corredor A., López-Cepero J., Fernández-López M., Mercado-Blanco J. (2021). The banana root endophytome: Differences between mother plants and suckers and evaluation of selected bacteria to control *Fusarium oxysporum* f.sp. cubense. J. Fungi.

[45] Montes-Osuna N., Gómez-Lama Cabanás C., Valverde-Corredor A., Berendsen R.L., Prieto P., Mercado-Blanco J. (2021). Assessing the involvement of selected phenotypes of *Pseudomonas simiae* PICF7 in olive root colonization and biological control of *Verticillium dahliae*. Plants.

[46] Mercado-Blanco J., Ramos J.-L., Goldberg J.B., Filloux A. (2015). Pseudomonas strains that exert biocontrol of plant pathogens. Pseudomonas.

[47] Patowary R., Deka H., Amaresan N., Senthil M.S., Kumar, Annapurna K., Kumar K., Sankaranarayanan A. (2020). Paenibacillus. Beneficial Microbes in Agro-Ecology.

[48] Khan M., Salman M., Jan S.A., Shinwari Z.K. (2021). Biological control of fungal phytopathogens: A comprehensive review based on *Bacillus* species. MOJ Biol. Med..

[49] Yu Y., Gui Y., Li Z., Jiang C., Guo J., Niu D. (2022). Induced systemic resistance for improving plant immunity by beneficial microbes. Plants.

[50] Kour D., Negi R., Sharief Khan S., Kumar S., Kaur S., Kaur T., Sharma B., Dasila H., Kour H., Ramniwas S. (2024). Microbes mediated induced systemic response in plants: A review. Plant Stress.

[51] Palaniyandi S.A., Yang S.H., Zhang L., Shu J.-V. (2013). Effects of actinobacteria on plant disease suppression and growth promotion. Appl. Microbiol. Biotechnol..

[52] Khan S., Srivastava S., Karnwal A., Malik T. (2023). *Streptomyces* as a promising biological control agents for plant pathogens. Front. Microbiol..

[53] Díaz-Díaz M., Antón-Domínguez B.I., Raya M.C., Bernal-Cabrera A., Medina-Marrero R., Trapero A., Agustí-Brisach C. (2024). *Streptomyces* spp. strains as potential biological control agents against Verticillium Wilt of Olive. J. Fungi.

[54] Alabouvette C., Olivain C., Migheli Q., Steinberg C. (2009). Microbiological control of soil-borne phytopathogenic fungi with special emphasis on wilt-inducing *Fusarium oxysporum*. New Phytol..

[55] Patil S., Sriram S. (2020). BiologicalI control of Fusarium wilt in crop plants using non-pathogenic isolates of *Fusarium* species. Indian Phytopathol..

[56] Ahmed M.E., Kararah M.A., Abada K.A., Eldakar H.A. (2023). Recent approaches for management of tomato fusarium wilt. Pak. J. Phytopathol..

[57] Çevik R., Demir S., Türkölmez Ş., Boyno G. (2022). The effect of *Clonostachys rosea* (sch.) schroers and samuels against verticillium wilt (*Verticillium dahliae* Kleb.) and early blight [*Alternaria solani* (Ell. and G. Martin) Sor.] diseases in tomato plants. Yuz. Yıl Univ. J. Agric. Sci..

[58] Rizk I.M., Mousa I.E., Ammar M.M., Abd-ElMaksoud I. (2017). Biological control of *Fusarium oxysporum* and *Verticillium dahliae* by *Trichoderma harzianum* and *Gliocladium virens* of two mint species. Res. J. Appl. Biotechnol..

[59] Boutaj H., Meddich A., Roche J., Mouzeyar S., El Modafar C. (2022). The effects of mycorrhizal fungi on vascular wilt diseases. Crop Prot..

[60] Pozo M.J., Azcón-Aguilar C. (2007). Unraveling mycorrhiza-induced resistance. Curr. Opin. Plant Biol..

[61] Villani A., Tommasi F., Paciolla C. (2021). The arbuscular mycorrhizal fungus *Glomus viscosum* improves the tolerance to verticillium wilt in artichoke by modulating the antioxidant defense systems. Cells.

[62] Mondal S., Halder S.K., Yadav A.N., Mondal K.C., Yadav A.N., Rastegari A.A., Yadav N., Kour D. (2020). Microbial consortium with multifunctional plant growth-promoting attributes: Future perspective in agriculture. Advances in Plant Microbiome and Sustainable Agriculture: Functional Annotation and Future Challenges.

[63] Prigigallo M.I., Gómez-Lama Cabanás C., Mercado-Blanco J., Bubici G. (2022). Designing a synthetic microbial community devoted to biological control: The case study of Fusarium wilt of banana. Front. Microbiol..

[64] Piombo E., Abdelfattah A., Droby S., Wisniewski M., Spadaro D., Schena L. (2021). Metagenomics approaches for the detection and surveillance of emerging and recurrent plant pathogens. Microorganisms.

[65] Fadiji A.E., Babalola O.O. (2020). Metagenomics methods for the study of plant-associated microbial communities: A review. J. Microbiol. Methods.

[66] Sarethy I.P., Saharan A. (2021). Genomics, proteomics and transcriptomics in the biological control of plant pathogens: A review. Indian Phytopathol..

[67] Kaushal M., Mahuku G., Swennen R. (2020). Metagenomic insights of the root colonizing microbiome associated with symptomatic and non-symptomatic bananas in Fusarium wilt infected fields. Plants.

[68] Doni F., Miranti M., Mispan M.S., Mohamed Z., Uphoff N. (2022). Multi-omics approaches for deciphering the microbial modulation of plants’ genetic potentials: What’s known and what’s next?. Rhizosphere.

[69] Martí J.M., Arias-Giraldo L.F., Díaz-Villanueva W., Arnau V., Rodríguez-Franco A., Garay C.P. (2020). Metatranscriptomic dynamics after *Verticillium dahliae* infection and root damage in *Olea europaea*. BMC Plant Biol..

[70] Khatabi B., Tabrizi N.M., Salekdeh G.H., Salekdeh G. (2016). Holistic sequencing: Moving forward from plant microbial proteomics to metaproteomics. Agricultural Proteomics.

[71] Salvato F., Kleiner M.A. (2024). Complete metaproteomic workflow for arabidopsis roots inoculated by synthetic bacteria. Methods Mol. Biol..

[72] Bertrand C., Gonzalez-Coloma A., Prigent-Combaret C. (2021). Plant metabolomics to the benefit of crop protection and growth stimulation. Advances in Botanical Research.

[73] Mashabela M.D., Piater L.A., Dubery I.A., Tugizimana F., Mhlongo M.I. (2022). Rhizosphere tripartite interactions and PGPR-mediated metabolic reprogramming towards ISR and plant priming: A metabolomics review. Biology.

[74] Hu X., Puri K.D., Gurung S., Klosterman S.J., Wallis C.M., Britton M., Durbin-Johnson B., Phinney B., Salemi M., Short D.P.G. (2019). Proteome and metabolome analyses reveal differential responses in tomato-*Verticillium dahliae*-interactions. J. Proteom..

[75] Cardoni M., Mercado-Blanco J. (2023). Confronting stresses affecting olive cultivation from the holobiont perspective. Front. Plant Sci..

[76] Berg G., Rybakova D., Grube M., Köberl M. (2016). The plant microbiome explored: Implications for experimental botany. J. Exp. Bot..

[77] Berg G., Rybakova D., Fischer D., Cernava T., Champomier Vergès M.-C., Charles T., Chen X., Cocolin L., Eversole K., Herrero Corral G. (2020). Microbiome definition re-visited: Old concepts and new challenges. Microbiome.

[78] Berg G., Köberl M., Rybakova D., Müller H., Grosch R., Smalla K. (2017). Plant microbial diversity is suggested as the key to future biocontrol and health trends. FEMS Microbial. Ecol..

[79] Liu Y.X., Qin Y., Bai Y. (2019). Reductionist synthetic community approaches in root microbiome research. Curr. Opin. Microbiol..

[80] Pérez-Lorente A.I., Romero D., Molina-Santiago C. (2024). Unravelling the impact of environmental factors in shaping plant microbiomes. Microb. Biotechnol..

[81] Berendsen R.L., Pieterse C.M., Bakker P.A. (2012). The rhizosphere microbiome and plant health. Trends Plant Sci..

[82] Olanrewaju O.S., Babalola O.O. (2022). The rhizosphere microbial complex in plant health: A review of interaction dynamics. J. Integr. Agric..

[83] Stone B.W., Weingarten E.A., Jackson C.R. (2018). The role of the phyllosphere microbiome in plant health and function. Annu. Plant Rev. Online.

[84] De Mandal S., Jeon J. (2023). Phyllosphere microbiome in plant health and disease. Plants.

[85] Mercado-Blanco J., Lugtenberg B.J.J. (2014). Biotechnological applications of bacterial endophytes. Curr. Biotechnol..

[86] Hardoim P.R., van Overbeek L.S., Berg G., Pirttilä A.M., Compant S., Campisano A., Döring M., Sessitsch A. (2015). The hidden world within plants: Ecological and evolutionary considerations for defining functioning of microbial endophytes. Microbiol. Mol. Biol. Rev..

[87] Kamoun S., Furzer O., Jones J.D., Judelson H.S., Ali G.S., Dalio R.J., Roy S.G., Schena L., Zambounis A., Panabières F. (2015). The top 10 oomycete pathogens in molecular plant pathology. Mol. Plant Pathol..

[88] Dey K.K., Ganguly S., Sahu J., Vaishnav A., Singh H.B. (2022). Plant–microbe interactions in the age of sequencing. Plant–Microbe Interactions.

[89] Nizamani M.M., Zhang Q., Muhae-Ud-Din G., Wang Y. (2023). High-throughput sequencing in plant disease management: A comprehensive review of benefits, challenges, and future perspectives. Phytopathol. Res..

[90] Arif I., Batool M., Schenk P.M. (2020). Plant microbiome engineering: Expected benefits for improved crop growth and resilience. Trends Biotechnol..

[91] Pascale A., Proietti S., Pantelides I.S., Stringlis I.A. (2020). Modulation of the root microbiome by plant molecules: The basis for targeted disease suppression and plant growth promotion. Front. Plant Sci..

[92] Lau S.-E., Teo W.F.A., Teoh E.Y., Tan B.C. (2022). Microbiome engineering and plant biostimulants for sustainable crop improvement and mitigation of biotic and abiotic stresses. Discov. Food.

[93] Hu H., Wang M., Huang Y., Xu Z., Xu P., Nie Y., Tang H. (2022). Guided by the principles of microbiome engineering: Accomplishments and perspectives for environmental use. mLife.

[94] Ayaz M., Li C.-H., Ali Q., Zhao W., Chi Y.-K., Shafiq M., Ali F., Yu X.-Y., Yu Q., Zhao J.-T. (2023). Bacterial and fungal biocontrol agents for plant disease protection: Journey from lab to field, current status, challenges, and global perspectives. Molecules.

[95] Zhao M., Zhao J., Yuan J., Hale L., Wen T., Huang Q., Vivanco J.M., Zhou J., Kowalchuk G.A., Shen Q. (2021). Root exudates drive soil-microbe-nutrient feedbacks in response to plant growth. Plant Cell Environ..

[96] Yang S., Liu H., Xie P., Wen T., Shen Q., Yuan J. (2023). Emerging pathways for engineering the rhizosphere microbiome for optimal plant health. J. Agric. Food Chem..

[97] Mendes L.W., Mendes R., Raaijmakers J.M., Tsai S.M. (2018). Breeding for soil-borne pathogen resistance impacts active rhizosphere microbiome of common bean. ISME J..

[98] Wen H.G., Zhao J.H., Zhang B.S., Gao F., Wu X.M., Yan Y.S., Zhang J., Guo H.S. (2023). Microbe-induced gene silencing boosts crop protection against soil-borne fungal pathogens. Nat. Plants..

[99] Pérez C.E., Cabral G.B., Aragão F.J. (2021). Host-induced gene silencing for engineering resistance to *Fusarium* in soybean. Plant Pathol..

[100] Shanmugam V., Sharma V., Bharti P., Jyoti P., Yadav S.K., Aggarwal R., Jain S. (2017). RNAi induced silencing of pathogenicity genes of *Fusarium* spp. for vascular wilt management in tomato. Ann. Microbiol..

[101] Schumann U., Smith N.A., Kazan K., Ayliffe M., Wang M.B. (2013). Analysis of hairpin RNA transgene-induced gene silencing in *Fusarium oxysporum*. Silence.

[102] Fan S., Zhou Y., Zhu N., Meng Q., Zhao Y., Xu J., Tang Y., Dai S., Yuan X. (2024). Exogenous Application of dsRNA—Inducing Silencing of the *Fusarium oxysporum Tup1* Gene and Reducing Its Virulence. Int. J. Mol. Sci..

[103] Sarwar A., Hassan M.N., Imran M., Iqbal M., Majeed S., Brader G., Sessitsch A., Hafeez F.Y. (2018). Biocontrol activity of surfactin A purified from *Bacillus* NH-100 and NH-217 against rice bakanae disease. Microbiol Res..

[104] Shi M., Chen L., Wang X.W., Zhang T., Zhao P.B., Song X.Y., Sun C.Y., Chen X.L., Zhou B.C., Zhang Y.Z. (2012). Antimicrobial peptaibols from *Trichoderma pseudokoningii* induce programmed cell death in plant fungal pathogens. Microbiology.

[105] Lay F.T., Brugliera F., Anderson M.A. (2003). Isolation and properties of floral defensins from ornamental tobacco and petunia. Plant Physiol..

[106] Gopinath V., Velusamy P. (2013). Extracellular biosynthesis of silver nanoparticles using *Bacillus* sp. GP-23 and evaluation of their antifungal activity towards *Fusarium oxysporum*. Spectrochim. Acta A Mol. Biomol. Spectrosc..

[107] Win T.T., Khan S., Fu P. (2020). Fungus (*Alternaria* sp.) mediated silver nanoparticles synthesis, characterization and application as phyto-pathogens growth inhibitor. J. Nanotechnol..

[108] Al-Zubaidi S., Al-Ayafi A., Abdelkader H. (2019). Biosynthesis, characterization and antifungal activity of silver nanoparticles by *Aspergillus Niger* isolate. J. Nanotechnol. Res..

[109] Sathiyabama M., Parthasarathy R. (2016). Biological preparation of chitosan nanoparticles and its in vitro antifungal efficacy against some phytopathogenic fungi. Carbohydr. Polym..

[110] Smaoui S., Ennouri K., Chakchouk-Mtibaa A., Sellem I., Bouchaala K., Karray-Rebai I., Mellouli L. (2018). Statistical versus artificial intelligence-based modeling for the optimization of antifungal activity against *Fusarium oxysporum* using *Streptomyces* sp. strain TN71. J. Mycol. Méd..

[111] Wang Y., Zou Q. (2024). Deep learning meta-analysis for predicting plant soil-borne fungal disease occurrence from soil microbiome data. Appl. Soil Ecol..

[112] Mumbanza F.M., Kiggundu A., Tusiime G., Tushemereirwe W.K., Niblett C., Bailey A. (2013). In vitro antifungal activity of synthetic dsRNA molecules against two pathogens of banana, *Fusarium oxysporum* f. sp. cubense and Mycosphaerella fijiensis. Pest Manag. Sci..

[113] Fernandes J.S., Angelo P.C.S., Cruz J.C., Santos J.M.M., Sousa N.R., Silva G.F. (2016). Post-transcriptional silencing of the *SGE1* gene induced by a dsRNA hairpin in *Fusarium oxysporum* f. sp. cubense, the causal agent of Panama disease. Genet. Mol. Res..

[114] Fei S., Constantin M., Peters J., Batley J., Aitken E., Mitter N. RNAi-based management for Fusarium wilt of banana. Proceedings of the International Symposia on Tropical and Temperate Horticulture—ISTTH2016.

[115] Pacheco R., Bonilla J., Paguay A., Magdama F., Chong P. (2024). In Vitro RNA-Mediated Gene Silencing of *Fusarium oxysporum* f. sp. *cubense* from Ecuador and Assessment of RNAi Molecule Stability in Banana Plants. PREPRINT (Version 1). https://www.researchsquare.com/article/rs-4837296/v1.

[116] Ghag S.B., Shekhawat U.K., Ganapathi T.R. (2012). Petunia floral defensins with unique prodomains as novel candidates for development of fusarium wilt resistance in transgenic banana plants. PLoS ONE.

[117] Atienza M.T.J.A., Magpantay M.D.A., Santos K.L.T., Mora N.B., Balaraman R.P., Gemeinhardt M.E., Dela Cueva F.M., Paterno E.S., Fernando L.M., Kohli P. (2021). Encapsulation of plant growth-promoting bacterial crude extract in nanoliposome and its antifungal property against *Fusarium oxysporum*. ACS Agr. Sci. Technol..

[118] Selvaraj M.G., Vergara A., Ruiz H., Safari N., Elayabalan S., Ocimati W., Blomme G. (2019). AI-powered banana diseases and pest detection. Plant Methods.

[119] Hayit T., Endes A., Hayit F. (2024). The severity level classification of Fusarium wilt of chickpea by pre-trained deep learning models. J. Plant Pathol..

[120] Hayit T., Endes A., Hayit F. (2024). KNN-based approach for the classification of fusarium wilt disease in chickpea based on color and texture features. Eur. J. Plant Pathol..

[121] Singh R.N., Krishnan P., Bharadwaj C., Das B. (2023). Improving prediction of chickpea wilt severity using machine learning coupled with model combination techniques under field conditions. Ecol. Inform..

[122] Naraghi L., Negahban M. (2020). Efficacy of *Talaromyces flavus* coated with nanoparticles in the growht inhibitory of *Fusarium oxysporum* f. sp. cucumerinum. 3c Tecnol. Glosas Innovación Apl. Pyme.

[123] Kaur A., Kukreja V., Aeri M., Tanwar S., Mohd N. Nature’s secrets revealed: Unraveling Fusarium wilt diseases through CNN and SVM. Proceedings of the 2023 4th IEEE Global Conference for Advancement in Technology (GCAT).

[124] Pareek M., Rajam M.V. (2017). RNAi-mediated silencing of MAP kinase signalling genes (*Fmk1, Hog1, and Pbs2*) in *Fusarium oxysporum* reduces pathogenesis on tomato plants. Fungal Biol..

[125] Bharti P., Jyoti P., Kapoor P., Sharma V., Shanmugam V., Yadav S.K. (2017). Host-induced silencing of pathogenicity genes enhances resistance to *Fusarium oxysporum* wilt in tomato. Mol. Biotechnol..

[126] Singh N., Mukherjee S.K., Rajam M.V. (2020). Silencing of the ornithine decarboxylase gene of *Fusarium oxysporum* f. sp. lycopersici by host-induced RNAi confers resistance to Fusarium wilt in tomato. Plant Mol. Biol. Rep..

[127] Tetorya M., Rajam M.V. (2021). RNAi-mediated silencing of *PEX6* and *GAS1* genes of *Fusarium oxysporum* f. sp. lycopersici confers resistance against Fusarium wilt in tomato. 3 Biotech.

[128] Chauhan S., Rajam M.V. (2022). RNAi-mediated down-regulation of fasciclin-like proteins (FoFLPs) in *Fusarium oxysporum* f. sp. lycopersici results in reduced pathogenicity and virulence. Microbiol. Res..

[129] Ouyang S.Q., Ji H.M., Feng T., Luo S.J., Cheng L., Wang N. (2023). Artificial trans-kingdom RNAi of FolRDR1 is a potential strategy to control tomato wilt disease. PLoS Pathog..

[130] Jo S.M., Ayukawa Y., Yun S.H., Komatsu K., Arie T. (2018). A putative RNA silencing component protein FoQde-2 is involved in virulence of the tomato wilt fungus *Fusarium oxysporum* f. sp. lycopersici. J. Gen. Plant Pathol..

[131] Ji H.M., Zhao M., Gao Y., Cao X.X., Mao H.Y., Zhou Y., Fan W.Y., Borkovich K.A., Ouyang S.Q., Liu P. (2018). *FRG3*, a target of slmiR482e-3p, provides resistance against the fungal pathogen *Fusarium oxysporum* in tomato. Front. Plant Sci..

[132] Ashraf H., Anjum T., Riaz S., Ahmad I.S., Irudayaraj J., Javed S., Qaiser U., Naseem S. (2021). Inhibition mechanism of green-synthesized copper oxide nanoparticles from *Cassia fistula* towards *Fusarium oxysporum* by boosting growth and defense response in tomatoes. Environ. Sci. Nano.

[133] Abdelraouf A.M.N., Hussain A.A., Naguib D.M. (2023). Nano-chitosan encapsulated *Pseudomonas fluorescens* greatly reduces Fusarium wilt infection in tomato. Rhizosphere.

[134] Ilyina A., Leon-Joublanc E., Balvantin-Garcia C., Montañez-Saenz J.C., Rodríguez-Garza M.M., Segura Ceniceros E.P., Martínez-Hernández J.L. (2013). Free and encapsulated chitinase and laminarinase as biological agents against *Fusarium oxysporum*. Afr. J. Microbiol. Res..

[135] Feng H., Gonzalez Viejo C., Vaghefi N., Taylor P.W., Tongson E., Fuentes S. (2022). Early detection of *Fusarium oxysporum* infection in processing tomatoes (*Solanum lycopersicum*) and pathogen–soil interactions using a low-cost portable electronic nose and machine learning modeling. Sensors.

[136] El-Sayed E.S.R., Mohamed S.S., Mousa S.A., El-Seoud M.A.A., Elmehlawy A.A., Abdou D.A. (2023). Bifunctional role of some biogenic nanoparticles in controlling wilt disease and promoting growth of common bean. AMB Express.

[137] do Prado E.V. (2022). Early detection of Fusarium wilt in common bean, at three levels of infestation, using leaf spectral information. Int. J. Adv. Eng. Manag..

[138] Castro-Valdecantos P., Egea G., Borrero C., Pérez-Ruiz M., Avilés M. (2024). Detection of Fusarium wilt-induced physiological impairment in strawberry plants using hyperspectral imaging and machine learning. Precis. Agric..

[139] Shinkado S., Saito H., Yamazaki M., Kotera S., Arazoe T., Arie T., Kamakura T. (2022). Genome editing using a versatile vector-based CRISPR/Cas9 system in *Fusarium* species. Sci. Rep..

[140] Mosa M.A., Youssef K. (2021). Topical delivery of host induced RNAi silencing by layered double hydroxide nanosheets: An efficient tool to decipher pathogenicity gene function of Fusarium crown and root rot in tomato. Physiol. Mol. Plant Pathol..

[141] Bilgili A., Bilgili A.V., Tenekeci M.E., Karadağ K. (2023). Spectral characterization and classification of two different crown root rot and vascular wilt diseases (*Fusarium oxysporum* f. sp. radicis lycopersici and Fusarium solani) in tomato plants using different machine learning algorithms. Eur. J. Plant Pathol..

[142] Hu Z., Parekh U., Maruta N., Trusov Y., Botella J.R. (2015). Down-regulation of *Fusarium oxysporum* endogenous genes by host-delivered RNA interference enhances disease resistance. Front. Chem..

[143] Wang Q., Cobine P.A., Coleman J.J. (2018). Efficient genome editing in *Fusarium oxysporum* based on CRISPR/Cas9 ribonucleoprotein complexes. Fungal Genet Biol..

[144] Wang Q., Coleman J.J. (2019). CRISPR/Cas9-mediated endogenous gene tagging in *Fusarium oxysporum*. Fungal Genet. Biol..

[145] Wagner T.A., Cai Y., Bell A.A., Puckhaber L.S., Magill C., Duke S.E., Liu J. (2020). RNAi suppression of CYP82D P450 hydroxylase, an enzyme involved in gossypol biosynthesis, enhances resistance to Fusarium wilt in cotton. J. Phytopathol..

[146] Sahayaraj K., Rajesh S., Rathi J.M. (2012). Silver nanoparticles biosynthesis using marine alga *Padina pavonica* (Linn.) and its microbicidal activity. Dig. J. Nanomater. Biostruct..

[147] Manganiello G., Nicastro N., Ortenzi L., Pallottino F., Costa C., Pane C. (2024). *Trichoderma* biocontrol performances against baby-lettuce Fusarium wilt surveyed by hyperspectral imaging-based machine learning and infrared thermography. Agriculture.

[148] Xu J., Wang X., Li Y., Zeng J., Wang G., Deng C., Guo W. (2018). Host-induced gene silencing of a regulator of G protein signalling gene (*Vd RGS 1*) confers resistance to Verticillium wilt in cotton. Plant Biotechnol. J..

[149] Oulad Ziane S., Imehli Z., El Alaoui Talibi Z., Ibnsouda Koraichi S., Meddich A., El Modafar C. (2024). Biocontrol of tomato Verticillium wilt disease by plant growth-promoting bacteria encapsulated in alginate extracted from brown seaweed. Int. J. Biol. Macromol..

[150] Shin M.Y., Viejo C.G., Tongson E., Wiechel T., Taylor P.W., Fuentes S. (2023). Early detection of Verticillium wilt of potatoes using near-infrared spectroscopy and machine learning modeling. Comput. Electron. Agric..

[151] Lizarazo I., Rodriguez J.L., Cristancho O., Olaya F., Duarte M., Prieto F. (2023). Identification of symptoms related to potato Verticillium wilt from UAV-based multispectral imagery using an ensemble of gradient boosting machines. Smart Agric. Technol..

[152] Ma R., Zhang N., Zhang X., Bai T., Yuan X., Bao H., He D., Sun W., He Y. (2024). Cotton Verticillium wilt monitoring based on UAV multispectral-visible multi-source feature fusion. Comput. Electron. Agric..

[153] Blekos K., Tsakas A., Xouris C., Evdokidis I., Alexandropoulos D., Alexakos C., Katakis S., Makedonas A., Theoharato C., Lalos A. (2021). Analysis, modeling and multi-spectral sensing for the predictive management of verticillium wilt in olive groves. J. Sens. Actuator Netw..

[154] López-Escudero F.J., Romero J., Bocanegra-Caro R., Santos-Rufo A. (2023). Predicting the risk of Verticillium wilt in olive orchards using Fuzzy Logic. Agriculture.

[155] Muramoto N., Tanaka T., Shimamura T., Mitsukawa N., Hori E., Koda K., Otani M., Hirai M., Nakamura K., Imaeda T. (2012). Transgenic sweet potato expressing thionin from barley gives resistance to black rot disease caused by *Ceratocystis fimbriata* in leaves and storage roots. Plant Cell Rep..

[156] Souza J.R., Mendes C.C., Guizilini V., Vivaldini K.C., Colturato A., Ramos F., Wolf D.F. Automatic detection of ceratocystis wilt in eucalyptus crops from aerial images. Proceedings of the 2015 IEEE International Conference on Robotics and Automation (ICRA).

[157] Wei X., Zhang J., Conrad A.O., Flower C.E., Pinchot C.C., Hayes-Plazolles N., Chen Z., Song Z., Fei S., Jin J. (2023). Machine learning-based spectral and spatial analysis of hyper-and multi-spectral leaf images for Dutch elm disease detection and resistance screening. Artif. Intell. Agric..

[158] Jiang G., Zhang Y., Gan G., Li W., Wan W., Jiang Y., Yang T., Zhang Y., Xu Y., Wang Y. (2022). Exploring rhizo-microbiome transplants as a tool for protective plant-microbiome manipulation. ISME Comm..

[159] Fernández-González A.J., Cardoni M., Gómez-Lama Cabanás C., Valverde-Corredor A., Villadas P.J., Fernández-López M., Mercado-Blanco J. (2020). Linking belowground microbial network changes to different tolerance level towards Verticillium wilt of olive. Microbiome.

[160] Kwak M.J., Kong H.G., Choi K., Kwon S.K., Song J.Y., Lee J., Lee P.A., Choi S.Y., Seo M., Lee H.J. (2018). Rhizosphere microbiome structure alters to enable wilt resistance in tomato. Nat. Biotechnol..

[161] Bziuk N., Maccario L., Sørensen S.J., Schikora A., Smalla K. (2022). Barley rhizosphere microbiome transplantation—A strategy to decrease susceptibility of barley grown in soils with low microbial diversity to powdery mildew. Front. Microbiol..

[162] Connell J.L., Ritschdorff E.T., Whiteley M., Shear J.B. (2013). 3D printing of microscopic bacterial communities. Proc. Natl. Acad. Sci. USA.

[163] Ke J., Wang B., Yoshikuni Y. (2021). Microbiome engineering: Synthetic biology of plant-associated microbiomes in sustainable agriculture. Trends Biotechnol..

[164] Massalha H., Korenblum E., Malitsky S., Shapiro O.H., Aharoni A. (2017). Live imaging of root-bacteria interactions in a microfluidics setup. Proc. Natl. Acad. Sci. USA.

[165] Massalha H., Korenblum E., Shapiro O.H., Aharoni A. (2019). Tracking root interactions system (TRIS) experiment and quality control. Bio. Protoc..

[166] Nezhad A.S. (2014). Microfluidic platforms for plant cells studies. Lab Chip.

[167] Jeong H.H., Jin S.H., Lee B.J., Kim T., Lee C.S. (2015). Microfluidic static droplet array for analyzing microbial communication on a population gradient. Lab Chip.

[168] Mohan R., Sanpitakseree C., Desai A.V., Sevgen S.E., Schroeder C.M., Kenis P.J. (2015). A microfluidic approach to study the effect of bacterial interactions on antimicrobial susceptibility in polymicrobial cultures. RSC Adv..

[169] Mandolini E., Probst M., Peintner U. (2021). Methods for studying bacterial–fungal interactions in the microenvironments of soil. App. Sci..

[170] Masters-Clark E., Clark A.J., Stanley C.E. (2022). Microfluidic tools for probing fungal-microbial interactions at the cellular level. JoVE.

[171] Orozco-Mosqueda M.d.C., Rocha-Granados M.d.C., Glick B.R., Santoyo G. (2018). Microbiome engineering to improve biocontrol and plant growth-promoting mechanisms. Microbiol. Res..

[172] Nandini B., Mawale K.S., Giridhar P. (2023). Nanomaterials in agriculture for plant health and food safety: A comprehensive review on the current state of agro-nanoscience. 3 Biotech..

[173] Boruah S., Dutta P. (2021). Fungus mediated biogenic synthesis and characterization of chitosan nanoparticles and its combine effect with *Trichoderma asperellum* against *Fusarium oxysporum*, *Sclerotium rolfsii* and *Rhizoctonia solani*. Indian Phytopathol..

[174] Das S., Pattanayak S., Das S.K. (2020). Nanotechnological approaches in sustainable agriculture and plant disease management. Organic Agriculture.

[175] Namasivayam S.K.R., Vinodhini R.K., Kavisri M., Bharani R.A., Moovendhan M. (2022). Formulation of biocontrol agents from *Trichoderma viride* and evaluation of viability, compatibility with metallic nanoparticles and decomposition efficacy of organic wastes. Biomass Convers. Bior..

[176] Ajilogba C.F., Babalola O.O., Nikoro D.O., Babalola O.O. (2021). Nanotechnology as vehicle for biocontrol of plant diseases in crop production. Food Security and Safety: African Perspectives.

[177] Kulkarni D., Sherkar R., Shirsathe C., Sonwane R., Varpe N., Shelke S., More M.P., Pardeshi S.R., Dhaneshwar G., Junnuthula V. (2023). Biofabrication of nanoparticles: Sources, synthesis, and biomedical applications. Front. Bioeng. Biotechnol..

[178] Omran B.A., Baek K.H. (2022). Control of phytopathogens using sustainable biogenic nanomaterials: Recent perspectives, ecological safety, and challenging gaps. J. Clean. Prod..

[179] Ahmed A., Usman M., Ji Z., Rafiq M., Yu B., Shen Y., Cong H. (2023). Nature-inspired biogenic synthesis of silver nanoparticles for antibacterial applications. Mater. Today Chem..

[180] Tomah A.A., Zhang Z., Alamer I.S.A., Khattak A.A., Ahmed T., Hu M., Wang D., Xu L., Li B., Wang Y. (2023). The potential of *Trichoderma*-mediated nanotechnology application in sustainable development scopes. Nanomaterials.

[181] Gajera H.P., Hirpara D.G., Bhadani R.V., Golakiya B.A. (2022). Green synthesis and characterization of nanosilver derived from extracellular metabolites of potent *Bacillus subtilis* for antifungal and eco-friendly action against phytopathogen. Biometals.

[182] Malik M.A., Wani A.H., Bhat M.Y., Siddiqui S., Alamri S.A., Alrumman S.A. (2024). Fungal-mediated synthesis of silver nanoparticles: A novel strategy for plant disease management. Front. Microbiol..

[183] Taheri P., Tarighi S., Ahmed F.K. (2024). The antagonistic yeasts: Novel nano/biofungicides for controlling plant pathogens. Nanohybrid Fungicides.

[184] Tauseef A., Uddin I., Uddin I. (2024). Novel Insights on Sustainable Nanoparticles in Crop Protection: Current Status and Future Prospectives. Sustainable Nanomaterials. Sustainable Materials and Technology.

[185] Ashraf H., Anjum T., Riaz S., Naseem S. (2020). Microwave-assisted green synthesis and characterization of silver nanoparticles using *Melia azedarach* for the management of Fusarium wilt in tomato. Front. Microbiol..

[186] Sankaranarayanan P., Anboli T.A., Suchithra T.V., Bose S., Shukla A.C., Baig M.R., Banerjee S. (2024). Agro-wastes-based feedstock as a source for bionanomaterials production: Outcomes and challenges. Concepts in Pharmaceutical Biotechnology and Drug Development.

[187] Mughal B., Zaidi S.Z.J., Zhang X., Hassan S.U. (2021). Biogenic nanoparticles: Synthesis, characterisation and applications. Appl. Sci..

[188] Dikshit P.K., Kumar J., Das A.K., Sadhu S., Sharma S., Singh S., Gupta P.K., Kim B.S. (2021). Green synthesis of metallic nanoparticles: Applications and limitations. Catalysts.

[189] Bandeira M., Giovanela M., Roesch-Ely M., Devine D.M., da Silva Crespo J. (2020). Green synthesis of zinc oxide nanoparticles: A review of the synthesis methodology and mechanism of formation. Sustain. Chem. Pharm..

[190] Abd El Aty A.A., Zohair M.M. (2020). Green-synthesis and optimization of an eco-friendly nanobiofungicide from *Bacillus amyloliquefaciens* MH046937 with antimicrobial potential against phytopathogens. Environ. Nanotechnol. Monit. Manag..

[191] Anjum S., Vyas A., Sofi T. (2023). Fungi-mediated synthesis of nanoparticles: Characterization process and agricultural applications. J. Sci. Food Agric..

[192] Ahmad A., Mukherjee P., Senapati S., Mandal D., Khan M.I., Kumar R., Sastry M. (2003). Extracellular biosynthesis of silver nanoparticles using the fungus *Fusarium oxysporum*. Colloids Surf. B Biointerfaces.

[193] Shiny K.S., Sundararaj R., Mamatha N., Lingappa B. (2019). A new approach to wood protection: Preliminary study of biologically synthesized copper oxide nanoparticle formulation as an environmental friendly wood protectant against decay fungi and termites. Maderas Cienc. Tecnol..

[194] Satti S.H., Raja N.I., Javed B., Akram A., Mashwani Z.-u.-R., Ahmad M.S., Ikram M. (2021). Titanium dioxide nanoparticles elicited agro-morphological and physicochemical modifications in wheat plants to control *Bipolaris sorokiniana*. PLoS ONE.

[195] Satti S.H., Raja N.I., Ikram M., Oraby H.F., Mashwani Z.U.R., Mohamed A.H., Singh A., Omar A.A. (2022). Plant-based titanium dioxide nanoparticles trigger biochemical and proteome modifications in *Triticum aestivum* L. under biotic stress of *Puccinia striiformis*. Molecules.

[196] Kumari A., Rana V., Yadav S.K., Kumar V. (2023). Nanotechnology as a powerful tool in plant sciences: Recent developments, challenges and perspectives. Plant Nano Biol..

[197] Karmous I., Vaidya S., Dimkpa C., Zuverza-Mena N., da Silva W., Barroso K.A., Milagres J., Bharadwaj A., Abdelraheem W., White J.C. (2023). Biologically synthesized zinc and copper oxide nanoparticles using *Cannabis sativa* L. enhance soybean (*Glycine max*) defense against *Fusarium virguliforme*. Pestic. Biochem. Physiol..

[198] Del Buono D., Di Michele A., Costantino F., Trevisan M., Lucini L. (2021). Biogenic ZnO nanoparticles synthesized using a novel plant extract: Application to enhance physiological and biochemical traits in maize. Nanomaterials.

[199] Wohlmuth J., Tekielska D., Čechová J., Baránek M. (2022). Interaction of the nanoparticles and plants in selective growth stages—Usual effects and resulting impact on usage perspectives. Plants.

[200] LewisOscar F., Vismaya S., Arunkumar M., Thajuddin N., Dhanasekaran D., Nithya C. (2016). Algal nanoparticles: Synthesis and biotechnological potentials. Algae-Organisms for Imminent Biotechnology.

[201] Yousefzadi M., Rahimi Z., Ghafori V. (2014). The green synthesis, characterization and antimicrobial activities of silver nanoparticles synthesized from green alga *Enteromorpha flexuosa* (wulfen) *J*. Agardh. Mat. Lett..

[202] Shankar P.D., Shobana S., Karuppusamy I., Pugazhendhi A., Ramkumar V.S., Arvindnarayan S., Kumar G. (2016). A review on the biosynthesis of metallic nanoparticles (gold and silver) using bio-components of microalgae: Formation mechanism and applications. Enzyme Microb. Technol..

[203] Kokabi M., Yousefzadi M., Ghorbanpour M., Shahid M.H. (2022). Algal nanoparticles and their potential application in agriculture. Nano-enabled Agrochemicals in Agriculture.

[204] Waqif H., Munir N., Farrukh M.A., Hasnain M., Sohail M., Abideen Z. (2024). Algal macromolecular mediated synthesis of nanoparticles for their application against citrus canker for food security. Int. J. Biol. Macromol..

[205] Abd-Elsalam K.A., Periakaruppan R., Rajeshkumar S. (2021). Agri-Waste and Microbes for Production of Sustainable Nanomaterials.

[206] T-Thienprasert N.P., Nattanan T., Jiraroj T., Ruangtong J., Jaithon T., Huehne P.S., Piasai O. (2021). Large scale synthesis of green synthesized zinc oxide nanoparticles from banana peel extracts and their inhibitory effects against *Colletotrichum* sp., isolate KUFC 021, causal agent of anthracnose on *Dendrobium orchid*. J. Nanomater..

[207] Goswami P., Mathur J. (2022). Application of agro-waste-mediated silica nanoparticles to sustainable agriculture. Bioresour. Bioprocess.

[208] Raliya R., Saharan V., Dimkpa C., Biswas P. (2018). Nanofertilizer for precision and sustainable agriculture: Current state and future perspectives. J. Agric. Food Chem..

[209] Keswani C., Bisen K., Singh V., Sarma B.K., Singh H.B., Arora N., Mehnaz S., Balestrini R. (2016). Formulation technology of biocontrol agents: Present status and future prospects. Bioformulations: For Sustainable Agriculture.

[210] Zainab R., Hasnain M., Ali F., Abideen Z., Siddiqui Z.S., Jamil F., Hussain M., Park Y.K. (2024). Prospects and challenges of nanopesticides in advancing pest management for sustainable agricultural and environmental service. Environ. Res..

[211] Hudson D., Margaritis A. (2014). Biopolymer nanoparticle production for controlled release of biopharmaceuticals. Crit. Rev. Biotechnol..

[212] Balla A., Silini A., Cherif-Silini H., Chenari Bouket A., Alenezi F.N., Belbahri L. (2022). Recent advances in encapsulation techniques of plant growth-promoting microorganisms and their prospects in the sustainable agriculture. Appl. Sci..

[213] Vemmer M., Patel A.V. (2013). Review of encapsulation methods suitable for microbial biological control agents. Biol. Control.

[214] Muñoz-Celaya A.L., Ortiz-García M., Vernon-Carter E.J., Jauregui-Rincón J., Galindo E., Serrano-Carreón L. (2012). Spray-drying microencapsulation of *Trichoderma harzianum* conidias in carbohydrate polymers matrices. Carbohydr. Polym..

[215] Pour M.M., Saberi-Riseh R., Mohammadinejad R., Hosseini A. (2019). Nano-encapsulation of plant growth-promoting rhizobacteria and their metabolites using alginate-silica nanoparticles and carbon nanotube improves UCB1 pistachio micropropagation. J. Microbiol. Biotechnol..

[216] Saberi-Riseh R., Hassanisaadi M., Vatankhah M., Soroush F., Varma R.S. (2022). Nano/microencapsulation of plant biocontrol agents by chitosan, alginate, and other important biopolymers as a novel strategy for alleviating plant biotic stresses. Int. J. Biol. Macromol..

[217] Saberi-Riseh R., Moradi-Pour M. (2021). A novel encapsulation of *Streptomyces fulvissimus* Uts22 by spray drying and its biocontrol efficiency against *Gaeumannomyces graminis*, the causal agent of take-all disease in wheat. Pest Manag. Sci..

[218] Nguyen M.H., Tran T.N.M., Vu N.B.D. (2021). Antifungal activity of essential oil-encapsulated lipid nanoemulsions formulations against leaf spot disease on tomato caused by *Alternaria alternata*. Arch. Phytopathol. Plant Protect..

[219] Russell S.J., Norvig P. (2010). Artificial Intelligence: A Modern Approach.

[220] Oxford (2019). Artificial Intelligence. Oxford Dict..

[221] Jordan M.I., Mitchell T.M. (2015). Machine learning: Trends, perspectives, and prospects. Science.

[222] Lecun Y., Bengio Y., Hinton G. (2015). Deep learning. Nature.

[223] Hirschberg J., Manning C.D. (2015). Advances in Natural Language Processing. Science.

[224] Wakchaure M., Patle B.K., Mahindrakar A.K. (2023). Application of AI techniques and robotics in agriculture: A review. Artif. Intell. Life Sci..

[225] Abiri R., Rizan N., Balasundram S.K., Shahbazi A.B., Abdul-Hamid H. (2023). Application of digital technologies for ensuring agricultural productivity. Heliyon.

[226] Mana A.A., Allouhi A., Hamrani A., Rahman S., el Jamaoui I., Jayachandran K. (2024). Sustainable AI-based production agriculture: Exploring AI applications and implications in agricultural practices. Smart Agric. Technol..

[227] Chen G., Pham T.T. (2000). Introduction to Fuzzy Sets, Fuzzy Logic, and Fuzzy Control Systems.

[228] Sadeghi M., Panahi B., Mazlumi A., Hejazi M.A., Komi D.E.A., Nami Y. (2022). Screening of potential probiotic lactic acid bacteria with antimicrobial properties and selection of superior bacteria for application as biocontrol using machine learning models. LWT.

[229] Liao J.R., Lee H.C., Chiu M.C., Ko C.C. (2020). Semi-automated identification of biological control agent using artificial intelligence. Sci. Rep..

[230] El-Naggar N.E.A., Bashir S.I., Rabei N.H., Saber W.I. (2022). Innovative biosynthesis, artificial intelligence-based optimization, and characterization of chitosan nanoparticles by *Streptomyces microflavus* and their inhibitory potential against *Pectobacterium carotovorum*. Sci. Rep..

[231] El-Naggar N.E., Sherief A.A., Hamza S.S. (2011). *Streptomyces aegyptia* NEAE 102, a novel cellulolytic streptomycete isolated from soil in Egypt. Afr. J. Microbiol. Res..

[232] Talib N.S.R., Halmi M.I.E., Gani S.S.A., Zaidan U.H., Shukor M.Y.A. (2019). Artificial neural networks (ANNs) and response surface methodology (RSM) approach for Modelling the optimization of chromium (VI) reduction by newly isolated *Acinetobacter radioresistens* strain NS-MIE from agricultural soil. BioMed Res. Int..

[233] Souza R., Armanhi J., Arruda P. (2020). From microbiome to traits: Designing synthetic microbial communities for improved crop resiliency. Front. Plant Sci..

[234] Martins S., Pasche J., Silva H., Selten G., Savastano N., Abreu L., Bais H., Garrett K., Kraisitudomsook N., Pieterse C. (2023). The use of synthetic microbial communities (SynComs) to improve plant health. Phytopathology.

[235] Wan T., Zhao H., Wang W. (2017). Effect of biocontrol agent *Bacillus amyloliquefaciens* SN16-1 and plant pathogen *Fusarium oxysporum* on tomato rhizosphere bacterial community composition. Biol. Control.

[236] Gómez-Lama Cabanás C., Wentzien N.M., Zorrilla-Fontanesi Y., Valverde-Corredor A., Fernández-González A.J., Fernández-López M., Mercado-Blanco J. (2022). Impacts of the biocontrol strain *Pseudomonas simiae* PICF7 on the banana holobiont: Alteration of root microbial co-occurrence networks and effect on host defense responses. Front Microbiol..

[237] Cardoni M., Fernández-González A.J., Valverde-Corredor A., Fernández-López M., Mercado-Blanco J. (2023). Co-occurrence network analysis unveils the actual differential impact on the olive root microbiota by two Verticillium wilt biocontrol rhizobacteria. Environ. Microbiome.

[238] Kotula H.J., Peralta G., Frost C.M., Todd J.H., Tylianakis J.M. (2021). Predicting direct and indirect non-target impacts of biocontrol agents using machine-learning approaches. PLoS ONE.

[239] Zhao L., Walkowiak S., Fernando W.G.D. (2023). Artificial intelligence: A promising tool in exploring the phytomicrobiome in managing disease and promoting plant health. Plants.

[240] Sperschneider J. (2020). Machine learning in plant–pathogen interactions: Empowering biological predictions from field scale to genome scale. New Phytol..

[241] Pane C., Manganiello G., Nicastro N., Ortenzi L., Pallottino F., Cardi T., Costa C. (2021). Machine learning applied to canopy hyperspectral image data to support biological control of soil-borne fungal diseases in baby leaf vegetables. Biol. Control.

[242] Boschert S., Rosen R., Hehenberger P., Bradley D. (2016). Digital twin—The simulation aspect. Mechatronic futures: Challenges and solutions for mechatronic systems and their designers. Mechatronic Futures.

[243] Grieves M., Vickers J., Kahlen J., Flumerfelt S., Alves A. (2017). Digital twin: Mitigating unpredictable, undesirable emergent behavior in complex systems. Transdisciplinary Perspectives on Complex Systems.

[244] Verdouw C., Tekinerdogan B., Beulens A., Wolfert S. (2021). Digital twins in smart farming. Agric. Syst..

[245] Xu Y., Li Z. (2020). CRISPR-Cas systems: Overview, innovations and applications in human disease research and gene therapy. Comput. Struct. Biotechnol. J..

[246] Miller J.C., Holmes M.C., Wang J., Guschin D.Y., Lee Y.L. (2007). An improved zinc-finger nuclease architecture for highly specific genome editing. Nat. Biotechnol..

[247] Christian M., Cermak T., Doyle E.L., Schmidt C., Zhang F. (2010). Targeting DNA double-strand breaks with TAL effector nucleases. Genetics.

[248] Westra E.R., Dowling A.J., Broniewski J.M., Houte S. (2016). Evolution and ecology of CRISPR. Annu. Rev. Ecol. Evol. Syst..

[249] Chen P.J., Liu D.R. (2023). Prime editing for precise and highly versatile genome manipulation. Nat. Rev. Genet..

[250] Chen Y.-H., Lu J., Yang X., Huang L.-C., Zhang C.-Q., Liu Q.-Q., Li Q.-F. (2023). Gene editing of non-coding regulatory DNA and its application in crop improvement. J. Exp. Bot..

[251] Gokul A., Mabaso J., Henema N., Otomo L., Bakare O.O., Klein A., Daniel A.I., Omolola A., Niekerk L.-A., Nkomo M. (2023). Sustainable agriculture through the enhancement of microbial biocontrol agents: Current challenges and new perspectives. Appl. Sci..

[252] Wang Q. (2019). Development of a CRISPR/Cas9 Gene Editing System for *Fusarium oxysporum* and Characterization of an Extracellular Superoxide Dismutase and Its Contribution to Pathogenicity on Cotton. Ph.D. Thesis.

[253] Yoshida T., Kawabe M., Miyata Y., Teraoka T., Arie T. (2008). Biocontrol activity in a nonpathogenic REMI mutant of *Fusarium oxysporum* f. sp. conglutinans and characterization of its disrupted gene. J. Pestic. Sci..

[254] Ghorbanpour M., Omidvari M., Abbaszadeh-Dahaji P., Omidvar R., Kariman K. (2018). Mechanisms underlying the protective effects of beneficial fungi against plant diseases. Biol. Control.

[255] Xu X., Huang R., Yin W.B. (2021). An optimized and efficient crispr/cas9 system for the endophytic fungus *Pestalotiopsis fici*. J. Fungi.

[256] Muñoz I.V., Sarrocco S., Malfatti L., Baroncelli R., Vannacci G. (2019). CRISPR-Cas for fungal genome editing: A new tool for the management of plant diseases. Front. Plant Sci..

[257] Chowdhary K., Arora H., Sharma S. (2022). CRISPR/Cas9-based genome editing as a way ahead for inducing production of bioactive metabolites in endophytes. Natl. Acad. Sci. Lett..

[258] Huang P.W., Yang Q., Zhu Y.L., Zhou J., Sun K., Mei Y.Z., Dai C.C. (2020). The construction of CRISPR-Cas9 system for endophytic *Phomopsis liquidambaris* and its PmkkA-deficient mutant revealing the effect on rice. Fungal Genet. Biol..

[259] Zhu Y.L., Zhang M.Q., Wang L.S., Mei Y.Z., Dai C.C. (2022). Overexpression of chitinase in the endophyte *Phomopsis liquidambaris* enhances wheat resistance to *Fusarium graminearum*. Fungal Genet. Biol..

[260] Yi Y., Li Z., Song C., Kuipers O.P. (2018). Exploring plant-microbe interactions of the rhizobacteria *Bacillus subtilis* and *Bacillus mycoides* by use of the CRISPR-Cas9 system. Environ. Microbiol..

[261] Urumbil S.K., Anilkumar M. (2021). Metagenomic insights into plant growth promoting genes inherent in bacterial endophytes of *Emilia sonchifolia* (Linn.) DC. Plant Sci. Today.

[262] Clouse K.M., Wagner M.R. (2021). Plant genetics as a tool for manipulating crop microbiomes: Opportunities and challenges. Front. Bioeng. Biotechnol..

[263] Anzalone A.V., Randolph P.B., Davis J.R., Sousa A.A., Koblan L.W., Levy J.M., Chen P.J., Wilson C., Newby G.A., Raguram A. (2019). Search-and-replace genome editing without double-strand breaks or donor DNA. Nature.

[264] Huang Z., Liu G. (2023). Current advancement in the application of prime editing. Front. Bioeng. Biotechnol..

[265] Ni P., Zhao Y., Zhou X., Liu Z., Huang Z., Ni Z., Sun O., Zong Y. (2023). Efficient and versatile multiplex prime editing in hexaploid wheat. Genome Biol..

[266] Zhao Z., Shang P., Mohanraju P., Geijsen N. (2023). Prime editing: Advances and therapeutic applications. Trends Biotechnol..

[267] Shelake R.M., Pramanik D., Kim J.-Y. (2019). Exploration of plant-microbe interactions for sustainable agriculture in CRISPR era. Microorganisms.

[268] Hassan M.M., Yuan G., Chen J.G., Tuskan G.A., Yang X. (2020). Prime editing technology and its prospects for future applications in plant biology research. Biodes. Res..

[269] Rajput M., Choudhary K., Kumar M., Vivekanand V., Chawade A., Ortiz R., Pareek N. (2021). RNA interference and CRISPR/Cas gene editing for crop improvement: Paradigm shift towards sustainable sgriculture. Plants.

[270] Parperides E., El Mounadi K., Garcia-Ruiz H. (2023). Induction and suppression of gene silencing in plants by nonviral microbes. Mol. Plant Pathol..

[271] Hudzik C., Hou Y., Ma W., Axtell M.J. (2020). Exchange of small regulatory RNAs between plants and their pests. Plant Physiol..

[272] Garcia-Ruiz H., Garcia Ruiz M.T., Gabriel Peralta S.M., Miravel Gabriel C.B., El-Mounadi K. (2016). Mecanismos, aplicaciones y perspectivas del silenciamiento génico de virus en plantas. Rev. Mex. Fitopatol..

[273] Rosa C., Kuo Y.W., Wuriyanghan H., Falk B.W. (2018). RNA interference mechanisms and applications in plant pathology. Annu. Rev. Phytopathol..

[274] Cai Q., Qiao L., Wang M., He B., Lin F.M., Palmquist J., Huang S.D., Jin H. (2018). Plants send small RNAs in extracellular vesicles to fungal pathogen to silence virulence genes. Science.

[275] Koch A., Biedenkopf D., Furch A., Weber L., Rossbach O., Abdellatef E., Linicus L., Johannsmeier J., Jelonek L., Goesmann A. (2016). An RNAi-based control of *Fusarium graminearum* infections through spraying of long dsRNAs involves a plant passage and is controlled by the fungal silencing machinery. PLoS Pathog..

[276] Wang M., Weiberg A., Lin F.M., Thomma B.P.H.J., Huang H.D., Jin H.L. (2016). Bidirectional cross-kingdom RNAi and fungal uptake of external RNAs confer plant protection. Nat. Plants.

[277] Nowara D., Gay A., Lacomme C., Shaw J., Ridout C., Douchkov D., Hensel G., Kumlehn J., Schweizer P. (2010). HIGS: Host-induced gene silencing in the obligate biotrophic fungal pathogen *Blumeria graminis*. Plant Cell.

[278] Ghag S.B., Shekhawat U.K., Ganapathi T.R. (2014). Host-induced post-transcriptional hairpin RNA-mediated gene silencing of vital fungal genes confers efficient resistance against Fusarium wilt in banana. Plant Biotechnol. J..

[279] Koch A., Kumar N., Weber L., Keller H., Imani J., Kogel K.H. (2013). Host-induced gene silencing of cytochrome P450 lanosterol C14a-demethylase-encoding genes confers strong resistance to *Fusarium* species. Proc. Natl. Acad. Sci. USA.

[280] Zulfiqar S., Farooq M.A., Zhao T., Wang P., Tabusam J., Wang Y., Xuan S., Zhao J., Chen X., Shen S. (2023). Virus-Induced Gene Silencing (VIGS): A powerful tool for crop improvement and its advancement towards epigenetics. Int. J. Mol. Sci..

[281] Zhang Y., Niu N., Li S., Liu Y., Xue C., Wang H., Liu M., Zhao J. (2023). Virus-Induced Gene Silencing (VIGS) in Chinese Jujube. Plants.

[282] Wang Z., Cao S., Xu X., He Y., Shou W., Munaiz E.D., Yu C., Shen J. (2023). Application and expansion of virus-induced gene silencing for functional studies in vegetables. Horticulturae.

[283] Liu C., Kogel K.H., Ladera-Carmona M. (2024). Harnessing RNA interference for the control of *Fusarium* species: A critical review. Mol. Plant Pathol..

[284] Gebremichael D.E., Haile Z.M., Negrini F., Sabbadini S., Capriotti L., Mezzetti B., Baraldi E. (2021). RNA interference strategies for future management of plant pathogenic fungi: Prospects and challenges. Plants.

[285] Hough J., Howard J.D., Brown S., Portwood D.E., Kilby P.M., Dickman M.J. (2022). Strategies for the production of dsRNA biocontrols as alternatives to chemical pesticides. Front. Bioeng. Biotechnol..

[286] Niño-Sánchez J., Chen L.H., De Souza J.T., Mosquera S., Stergiopoulos I. (2021). Targeted delivery of gene silencing in fungi using genetically engineered bacteria. J. Fungi.

[287] Jiang Y., Liu X., Tian X., Zhou J., Wang Q., Wang B., Yu W., Jiang Y., Hsiang T., Qi X. (2023). RNA interference of *Aspergillus flavus* in response to *Aspergillus flavus* partitivirus 1 infection. Front. Microbiol..

[288] Mahanty B., Mishra R., Joshi R.K. (2023). Cross-kingdom small RNA communication between plants and fungal phytopathogens-recent updates and prospects for future agriculture. RNA Biol..

[289] Ray P., Sahu D., Aminedi R., Chandran D. (2022). Concepts and considerations for enhancing RNAi efficiency in phytopathogenic fungi for RNAi-based crop protection using nanocarrier-mediated dsRNA delivery systems. Front. Fungal Biol..

[290] Ghosh S., Patra S., Ray S. (2023). A Combinatorial nanobased spray-induced gene silencing technique for crop protection and improvement. ACS Omega.

[291] Rodríguez Melo J., Mammarella F., Ariel F. (2023). Exogenous RNAs: Promising tools for the second green revolution. J. Exp. Bot..

[292] Nagata T., Okada K., Takebe R., Matsui C. (1981). Delivery of tobacco mosaic virus RNA into plant protoplasts mediated by reverse-phase evaporation vesicles (liposomes). Mol. Genet. Genom..

[293] Silva A.T., Nguyen A., Ye C., Verchot J., Moon J.H. (2010). Conjugated polymer nanoparticles for effective siRNA delivery to tobacco BY-2 protoplasts. BMC Plant Biol..

[294] Liu J., Li R., Yang B. (2020). Carbon dots: A new type of carbon-based nanomaterial with wide applications. ACS Cent. Sci..

[295] Mitter N., Worrall E.A., Robinson K.E., Li P., Jain R.G., Taochy C., Fletcher S.J., Carroll B.J., Lu G.Q., Xu Z.P. (2017). Clay nanosheets for topical delivery of RNAi for sustained protection against plant viruses. Nat. Plants.

[296] Yan M., Yang C., Huang B., Huang Z., Zeqian L., Zhang X., Zhao C. (2017). Systemic toxicity induced by aggregated layered double hydroxide nanoparticles. Int. J. Nanomed..

[297] Ding T., Lin K., Chen J., Hu Q., Yang B., Li J., Gan J. (2018). Causes and mechanisms on the toxicity of layered double hydroxide (LDH) to green algae *Scenedesmus quadricauda*. Sci. Total Environ..

[298] Bennett M., Deikman J., Hendrix B., Iandolino A. (2020). Barriers to efficient foliar uptake of dsRNA and molecular barriers to dsRNA activity in plant cells. Front. Plant Sci..

[299] Das P.R., Sherif S.M. (2020). Application of exogenous dsRNAs-induced RNAi in agriculture: Challenges and triumphs. Front. Plant Sci..

[300] Šečić E., Kogel K.H. (2021). Requirements for fungal uptake of dsRNA and gene silencing in RNAi-based crop protection strategies. Curr. Opin. Biotechnol..

[301] Niu D., Hamby R., Sanchez J.N., Cai Q., Yan Q., Jin H. (2021). RNAs—A new frontier in crop protection. Curr. Opin. Biotechnol..

[302] Hoang B., Fletcher S.J., Brosnan C.A., Ghodke A.B., Manzie N., Mitter N. (2022). RNAi as a foliar spray: Efficiency and challenges to field applications. Int. J. Mol. Sci..

[303] Dou T., Shao X., Hu C., Liu S., Sheng O., Bi F., Deng G., Ding L., Li C., Dong T. (2020). Host-induced gene silencing of Foc TR4 *ERG6/11* genes exhibits superior resistance to Fusarium wilt of banana. Plant Biotechnol. J..

[304] Tetorya M., Rajam M.V. (2018). RNA silencing of *PEX 6* gene causes decrease in pigmentation, sporulation and pathogenicity of *Fusarium oxysporum*. Plant Pathol..

[305] Chauhan S., Rajam M.V. (2024). Host RNAi-mediated silencing of *Fusarium oxysporum* f. sp. lycopersici specific-fasciclin-like protein genes provides improved resistance to Fusarium wilt in Solanum lycopersicum. Planta.

[306] Song Y., Thomma B.P. (2018). Host-induced gene silencing compromises Verticillium wilt in tomato and Arabidopsis. Mol. Plant Pathol..

[307] Wei C., Qin T., Li Y., Wang W., Dong T., Wang Q. (2020). Host-induced gene silencing of the acetolactate synthases VdILV2 and VdILV6 confers resistance to Verticillium wilt in cotton (*Gossypium hirsutum* L.). Biochem. Biophys. Res. Commun..

[308] Su X., Lu G., Li X., Rehman L., Liu W., Sun G., Guo H., Wang G., Cheng H. (2020). Host-induced gene silencing of an adenylate kinase gene involved in fungal energy metabolism improves plant resistance to *Verticillium dahliae*. Biomolecules.

[309] Qiao L., Lan C., Capriotti L., Ah-Fong A., Nino Sanchez J., Hamby R., Heller J., Zhao H., Glass N.L., Judelson H.S. (2021). Spray-induced gene silencing for disease control is dependent on the efficiency of pathogen RNA uptake. Plant Biotechnol. J..

[310] Zhang T., Jin Y., Zhao J.H., Gao F., Zhou B.J., Fang Y.Y., Gou H.S. (2016). Host-induced gene silencing of the target gene in fungal cells confers effective resistance to the cotton wilt disease pathogen *Verticillium dahliae*. Mol. Plant.

[311] Li J., Hu S., Jian W., Xie C., Yang X. (2021). Plant antimicrobial peptides: Structures, functions, and applications. Bot. Stud..

[312] Montesinos E. (2023). Functional peptides for plant disease control. Annu. Rev. Phytopathol..

[313] Cavallarin L., Andreu D., San Segundo B. (1998). Cecropin A-derived peptides are potent inhibitors of fungal plant pathogens. Mol. Plant-Microbe Interact..

[314] Das K., Datta K., Karmakar S., Datta S.K. (2019). Antimicrobial peptides—Small but mighty weapons for plants to fight phytopathogens. Protein Pept Lett..

[315] Zhang Y.M., Ye D.X., Liu Y., Zhang X.Y., Zhou Y.L., Zhang L., Yang X.L. (2023). Peptides, new tools for plant protection in eco-agriculture. Adv. Agrochem..

[316] Pereira-Dias L., Oliveira-Pinto P.R., Fernandes J.O., Regalado L., Mendes R., Teixeira C., Mariz-Ponte N., Gomes P., Santos C. (2023). Peptaibiotics: Harnessing the potential of microbial secondary metabolites for mitigation of plant pathogens. Biotechnol. Adv..

[317] Pelegrini P.B., Noronha E.F., Muniz M.A.R., Vasconcelos I.M., Chiarello M.D., Oliveira J.T.A., Franco O.L. (2006). An antifungal peptide from passion fruit (*Passiflora edulis*) seeds with similarities to 2S albumin proteins. Biochim. Biophys. Acta Proteins Proteom..

[318] Cândido Ede S., Pinto M.F., Pelegrini P.B., Lima T.B., Silva O.N., Pogue R., Grossi-de-Sá M.F., Franco O.L. (2011). Plant storage proteins with antimicrobial activity: Novel insights into plant defense mechanisms. FASEB J..

[319] de Azevedo dos Santos L., Taveira G.B., da Silva M.S., da Silva Gebara R., da Silva Pereira L., Perales J., Teixeira-Ferreira A., de Oliveira Mello É., de Oliveira Carvalho A., Rodrigues R. (2020). Antimicrobial peptides from *Capsicum chinense* fruits: Agronomic alternatives against phytopathogenic fungi. Biosci. Rep..

[320] Turrini A., Sbrana C., Pitto L., Ruffini Castiglione M., Giorgetti L., Briganti R., Bracci T., Evangelista M., Nuti M.P., Giovannetti M. (2004). The antifungal Dm-AMP1 protein from *Dahlia merckii* expressed in *Solanum melongena* is released in root exudates and differentially affects pathogenic fungi and mycorrhizal symbiosis. New Phytol..

[321] Van Der Weerden N.L., Lay F.T., Anderson M.A. (2008). The plant defensin, NaD1, enters the cytoplasm of *Fusarium oxysporum* hyphae. J. Biol. Chem..

[322] Van Der Weerden N.L., Hancock R.E., Anderson M.A. (2010). Permeabilization of fungal hyphae by the plant defensin NaD1 occurs through a cell wall-dependent process. J. Biol. Chem..

[323] Rogozhin E.A., Oshchepkova Y.I., Odintsova T.I., Khadeeva N.V., Veshkurova O.N., Egorov T.A., Grishin E.V., Salikhov S.I. (2011). Novel antifungal defensins from *Nigella sativa* L. seeds. Plant Physiol. Biochem..

[324] Singh S. (2020). Investigation on the Role of Plant Defensin Proteins in Regulating Plant-*Verticillium longisporum* Interactions in *Arabidopsis thaliana*. Ph.D. Thesis.

[325] Gao X., Ding J., Liao C., Xu J., Liu X., Lu W. (2021). Defensins: The natural peptide antibiotic. Adv. Drug Deliv. Rev..

[326] Naguib D.M., Alzandi A.A., Shamkh I.M., Reyad N.E.H.A. (2021). Fabatin induce defense-related enzymes in cucumber against soil born pathogen, *Fusarium oxysporum*. Rhizosphere.

[327] Leannec-Rialland V., Atanasova V., Chereau S., Tonk-Rügen M., Cabezas-Cruz A., Richard-Forget F. (2022). Use of defensins to develop eco-friendly alternatives to synthetic fungicides to control phytopathogenic fungi and their mycotoxins. J. Fungi.

[328] Chan Y.L., Prasad V., Sanjaya, Chen K.H., Liu P.C., Chan M.T., Cheng C.P. (2005). Transgenic tomato plants expressing an *Arabidopsis* thionin (Thi2. 1) driven by fruit-inactive promoter battle against phytopathogenic attack. Planta.

[329] Berrocal-Lobo M., Segura A., Moreno M., López G., García-Olmedo F., Molina A. (2002). Snakin-2, an antimicrobial peptide from potato whose gene is locally induced by wounding and responds to pathogen infection. Plant Physiol..

[330] Tang R., Tan H., Dai Y., Li L., Huang Y., Yao H., Cai Y., Yu G. (2023). Application of antimicrobial peptides in plant protection: Making use of the overlooked merits. Front Plant Sci..

[331] Sweany R.R., Cary J.W., Jaynes J.M., Rajasekaran K. (2023). Broad-spectrum antimicrobial activity of synthetic peptides GV185 and GV187. Plant Dis..

[332] Baró A., Mora I., Montesinos L., Montesinos E. (2020). Differential susceptibility of *Xylella fastidiosa* strains to synthetic bactericidal peptides. Phytopathology.

[333] Güell I., Cabrefiga J., Badosa E., Ferre R., Talleda M., Bardají E., Planas M., Feliu L., Montesinos E. (2011). Improvement of the efficacy of linear undecapeptides against plant-pathogenic bacteria by incorporation of D-amino acids. Appl. Environ. Microbiol..

